# Periostin shows increased evolutionary plasticity in its alternatively spliced region

**DOI:** 10.1186/1471-2148-10-30

**Published:** 2010-01-28

**Authors:** Sebastian Hoersch, Miguel A Andrade-Navarro

**Affiliations:** 1 Bioinformatics and Computing Core, Koch Institute for Integrative Cancer Research, Massachusetts Institute of Technology, 77 Massachusetts Avenue, Cambridge, MA 02139, USA; 2 Bioinformatics Group, Max Delbrück Center for Molecular Medicine, Robert-Rössle-Strasse. 10, 13125 Berlin, Germany; 3 Computational Biology and Data Mining Group, Max Delbrück Center for Molecular Medicine, Robert-Rössle-Strasse. 10, 13125 Berlin, Germany

## Abstract

**Background:**

Periostin (POSTN) is a secreted extracellular matrix protein of poorly defined function that has been related to bone and heart development as well as to cancer. In human and mouse, it is known to undergo alternative splicing in its C-terminal region, which is devoid of known protein domains. Differential expression of periostin, sometimes of specific splicing isoforms, is observed in a broad range of human cancers, including breast, pancreatic, and colon cancer. Here, we combine genomic and transcriptomic sequence data from vertebrate organisms to study the evolution of periostin and particularly of its C-terminal region.

**Results:**

We found that the C-terminal part of periostin is markedly more variable among vertebrates than the rest of periostin in terms of exon count, length, and splicing pattern, which we interpret as a consequence of neofunctionalization after the split between periostin and its paralog transforming growth factor, beta-induced (TGFBI). We also defined periostin's sequential 13-amino acid repeat units - well conserved in teleost fish, but more obscure in higher vertebrates - whose secondary structure is predicted to be consecutive beta strands. We suggest that these beta strands may mediate binding interactions with other proteins through an extended beta-zipper in a manner similar to the way repeat units in bacterial cell wall proteins have been reported to bind human fibronectin.

**Conclusions:**

Our results, obtained with the help of the increasingly large collection of complete vertebrate genomes, document the evolutionary plasticity of periostin's C-terminal region, and for the first time suggest a basis for its functional role.

## Background

Periostin (POSTN, PN, OSF-2) is a secreted extracellular matrix (ECM) glycoprotein of up to 93 kDa with a role in cell adhesion. It was originally identified in cells of mesenchymal lineage - osteoblasts, osteoblast-derived cell lines, the periosteum, and the periodontal ligament [1,2]. Its role in the development of bones, teeth, and cartilage has been documented subsequently (e.g. [3-7]). Furthermore, periostin has in recent years prominently emerged as important on two distinct fronts, both of them with notable clinical implications: One is its differential expression in a wide array of epithelial tumors compared to their respective normal tissues (for reviews, see [8,9]). For the majority of cancers investigated, periostin expression was found to be increased over normal tissue, but there are distinct exceptions where this pattern is reversed. The other relevant area is its expression in the developing and the diseased heart (for reviews, see [10-13]). In the developing heart, periostin has been found to be expressed in distinct substructures. In pathological heart conditions, periostin expression has been described both in the context of acute events (myocardial infarction) [14,15], as well as chronic pathological conditions (pressure overload) [15,16].

A comprehensive understanding of periostin's functional spectrum is still actively developing, but certain core aspects emerging from those three major areas (skeletal development, heart development and disease, and cancer) are coming increasingly into focus. Studies have associated periostin with epithelial-mesenchymal transition (EMT) in cancer [17,18] and with mesenchymal differentiation [19-22] in the developing heart.

Early characterizations of periostin as an adhesion protein on the basis of its apparent homology to insect fasciclin [23] have subsequently been refined by expanding the collection of known ECM binding partners (see below), and secondly by illuminating functional aspects consistent with a role in contact-signaling [12]. Periostin protein, whose expression has been found to be promoted for example by TGFbeta1, 2, or 3 and BMP-2 in multiple studies [2,21,24-28], can bind to certain integrin receptors, which subsequently activate the Akt/PKB pathway via FAK and PI3K, leading to an enhanced migratory or invasive phenotype [14,29-33].

**Figure 1:**
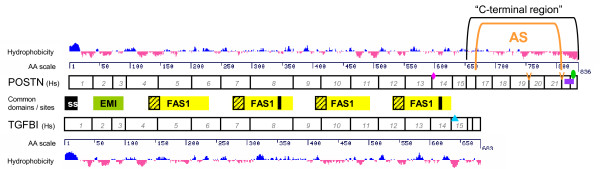
**Periostin and TGFBI exon and domain structure**. Numbered periostin (POSTN) and TGFBI exons (coding sequence only) are displayed to scale. Amino acid (AA) position scales and hydrophobicity profiles (adapted from the UCSC proteome browser [84]) for either protein are displayed above and below the exon structures, respectively. Domains and features in common are displayed in-between: a signal sequence (ss), an EMI domain, and four FAS1 domains. Vertical black bars in FAS1 domains 2 and 4 mark the integrin binding sites with conserved central DI dimers. The shaded regions in the N-terminal end of the FAS1 domains represent γ-glutamylcarboxylase recognition sites. Additional features specific to either of the two proteins are indicated by markers positioned in the respective exon structure as follows. Pink diamond: POSTN N-glycosylation site; blue triangle: TGFBI integrin RGD binding site; green oval: POSTN heparin binding motif (suspected); purple rectangle: POSTN bipartite nuclear localization signal. The two orange V's mark sites of genomic variation in periostin of other tetrapods: between exons 19 and 20, a cluster of 8 additional exon 19 copies observed in *X. tropicalis*, and an additional exon "21V22" between exons 21 and 22, observed for example in chicken. The periostin region between exons 16 and 22 is flagged as subject to widespread alternative splicing ("AS"), and the extent of the "C-terminal region" as referred to in this work is indicated. See the main text for detailed descriptions.

The periostin gene in human and mouse has 23 exons, with a genomic footprint covering about 36 (or 30) kilobases in human (or mouse). Both terminal exons in mouse and human are protein-coding. The periostin protein (see Figure 1) has an N-terminal signal sequence in accordance with its status as a secreted protein, an EMI domain, and four Fasciclin (FAS1) domains. The EMI domain, encoded by exons 2 and 3, is thought to be involved in protein-protein interactions or protein multimerization [34], and may be responsible for periostin dimers observed in some studies (e.g. [31,35]). A very recent report described EMI domain-mediated dimers of periostin and its paralog TGFBI (see below) [36], making it likely that the dimers observed in various periostin studies are indeed physiologically relevant.

Besides integrins, periostin has been described to bind a number of other ECM proteins, for example heparin [37], fibronectin [38], and collagen I [35,39], although the precise binding mechanisms are not defined in these cases. The four fasciclin (FAS1) domains, described as a cell adhesion module [23], are encoded by exons 3 to 14. As recently reported, each of these FAS1 domains contains an N-terminal recognition site for γ-glutamylcarboxylase, which mediates the post-translational modification of glutamate to γ-carboxyglutamate [40]. Also, integrin binding motifs are found in the second and fourth FAS1 domain, as inferred from findings of the periostin paralog transforming growth factor, beta-induced (TGFBI, also BIG-H3 or betaig-h3) [41].

Finally, exon 15 is followed by exons 16 - 23, making up 182 amino acids (in human) and thus a rather substantial part of the protein. The function of this stretch, sometimes referred to descriptively as "hydrophilic region" or, as from here onwards, "C-terminal region", is essentially unknown, although some aspects of its potential role have been investigated [42]. It is devoid of known domains and contains few known sequence motifs: (overlapping) regions of low compositional complexity and of intrinsic disorder (obviously interdependent features) and a C-terminal nuclear localization signal [43], which appears at odds with periostin's status as a secreted protein.

Interestingly, alternative splicing was described early on for human and murine periostin exclusively in this C-terminal region [1,2]. This is also suggested by EST sequence data for human or mouse periostin, where exons 17 to 21 present themselves as cassette exons that can be excluded from mature RNA message in various combinations, or individually.

The periostin paralog TGFBI is a protein with a domain structure identical to periostin (Figure 1) with the following exception: TGFBI, comprising only 17 exons, is notably shorter and lacks completely the C-terminal region that is subject to alternative splicing in periostin. Periostin and TGFBI sequences diverge fundamentally after the fourth fasciclin domain (i.e. starting at exon 15). An Arg-Gly-Asp (RGD) binding motif for a subgroup of integrin adhesion receptors (reviewed in [44]) is found in TGFBI exon 15, which is followed by two short exons 16 and terminal exon 17. Interestingly, TGFBI α3β1-integrin binding has been shown to be mediated not via the canonical RGD binding motif, but via two pentapeptides containing a central Asp-Ile (DI) dimer found in the second and fourth FAS1 domain [41], which were subsequently found to be conformationally similar to RGD peptides [45]. While an RGD motif is not present in periostin, the DI dimers in FAS1 domains 2 and 4 are conserved between the two proteins, strongly suggesting a mechanism for periostin integrin binding.

Much of the literature on TGFBI is dedicated to certain relatively common mutations causing a variety for corneal dystrophies (reviewed e.g. in [46]), a functional aspect for which there is no known counterpart for periostin. Like periostin, TGFBI is a TGFbeta-induced, secreted, integrin-binding ECM protein expressed during cardiac development and differentially expressed in certain epithelial cancers (e.g. [47]). But there is also mounting evidence that periostin and TGFBI can have complementary or opposite roles in cancer and development. For cancer, reports highlight a tumor-suppressive role of TGFBI (e.g. [48,49]), and complementary expression patterns of periostin and TGFBI in the developing heart were explicitly studied [50].

Here we present the results of our computational analysis of periostin's C-terminal region (exons 16 - 23), which constitutes the most visible difference between periostin and its paralog TGFBI and is hence likely to be a major determinant of the functional differences between these two genes and of periostin function per se.

Against the backdrop of unavailable functional or structural annotation and of indications for greater phylogenetic variability relative to the much better annotated rest of the gene, our interest was increased by the pervasive alternative splicing specific to this part of periostin. Periostin isoform heterogeneity is a source of significant complexity in the rapidly growing body of research on this gene, exacerbated by the fact that the precise nature of the variants observed or used experimentally is not always readily apparent. But cell-specific periostin isoform profiles have been demonstrated early on [2], and isoform-specific biological properties have been documented subsequently (e.g. [14,51,52]). It is hence not an exaggeration to expect that an improved understanding of periostin's C-terminal region in general and its alternative splicing patterns in particular may help resolve the sometimes controversial findings on periostin's biological effects.

## Methods

### Profile-based homology search

We compiled an alignment of two consecutive repeats from fragment 675-700 of periostin isoform 1 [*Danio rerio*] (RefSeq:NP_001071254.1) with fragments from another two sequences from *D. rerio *and one from *Xenopus tropicalis *found via BLAST [53]. The alignment was done using ClustalW [54] and manual editing. This alignment was converted into a profile and used for a search against UniRef100 with the program hmmsearch [55]. Iterative searches and addition of new positives led to a progressively refined profile with repeats from *Tetraodon nigroviridis*, *Takifugu rubripes*, and chicken, which matched mammalian periostin sequences (including human) at E-value 1.8 (3rd iteration). No false positive was observed below this E-value.

### Periostin locus identification and sequence reconstruction (assembled genomes)

Periostin loci in genome assemblies of interest (detailed in Additional file 1, Table S1) were identified in the UCSC genome browser [56] (accessible at http://genome.ucsc.edu/) via a combination of annotation tracks [57], where possible. Otherwise, the BLAT [58] search function within the genome browser was used to search with known periostin sequences from other organisms.

If available, transcript data (UCSC tracks '(species) mRNAs from GenBank' and '(species) ESTs That Have Been Spliced' were used to synthesize full-length periostin sequences with a complete set of exons, commonly resulting in full-length sequences with a higher exon count than found in available RefSeq sequences (Mm, Gg, Xt, Dr).

We identified individual missing exons or refined the boundaries of individual exons with the help of BLAT-based alignments and the 'Vertebrate Multiz Alignment & Conservation track' [59], which provides sequence-level genomic alignments for many vertebrate genomes. For teleosts, we used periostin gene predictions from the Ensembl database [60] (accessible at http://www.ensembl.org ), if available, in combination with transcript evidence or BLAST-based efforts to expand exon coverage.

For genome assemblies with insufficient or no transcript coverage of periostin, we performed TBLASTN [53] searches with a known full-length periostin sequence (usually human or chicken periostin for tetrapods and zebrafish locus 1 periostin for teleosts) as query against a genomic sequence fragment comprising the periostin locus as a subject sequence, changing default BLAST parameters to not mask repeats and to report hits with E-values higher than 10.0. TBLASTN hits were then evaluated in an exon-by-exon fashion: exact exon boundaries were defined aided by the 'Vertebrate Multiz Alignment & Conservation track' and on the basis of assuming splice site conservation with canonical GT-AG intronic splice sites in all cases as common practice in the comparative genomics field [61], and the correct sequential order of the exons in the genomic sequences was ascertained. It is hence important to bear in mind the putative nature of this group of predicted periostin sequences.

### Multiple sequence alignments and phylogenetic trees

All multiple sequence alignments were performed using the ClustalW algorithm as implemented in the ClustalX (version 2.0.11) software package [54], using default parameters, in particular using Neighbor Joining as the clustering algorithm. Bootstrapping values were obtained based on 1000 trials. Alignment-based phylogenetic trees were generated with gapped positions excluded and visualized with NJplot (described in [62]).

### Genomic sequence alignments

In addition to the seven tetrapod and five teleost species listed in Additional file 1, Table S1, we selected 16 additional species to study periostin exon 21V22 in the context of an alignment of genomic sequences containing the periostin locus.

Five additional genomic sequences were obtained from the UCSC genome browser [56] for dog (*Canis familiaris*, assembly canFam2), cow (*Bos taurus*, assembly bosTau4), horse (*Equus caballus*, assembly equCab2), zebra finch (*Taeniopygia guttata*, assembly taeGut1), and tentatively for lamprey (*Petromyzon marinus*, assembly petMar1).

Another 11 genomic sequences were obtained with the help of the Ensembl genome browser (http://www.ensembl.org) from generally less mature genome assemblies of other organisms, after the periostin locus was identified using the 'Projected human gene' track. These organisms were: armadillo (*Dasypus novemcinctus*), dolphin (*Tursiops truncates*), European hedgehog (*Erinaceus europaeus*), hyrax (*Procavia capensis*), kangaroo rat (*Dipodomys ordii*), lesser hedgehog tenrec (*Echinops telfairi*), megabat (*Pteropus vampyrus*), microbat (*Myotis lucifugus*), squirrel (*Spermophilus tridecemlineatus*), tarsier (*Tarsius syrichta*), and tree shrew (*Tupaia belangeri*).

The genome sequence alignments were then performed using the VISTA suite of computational genomics web tools [63] (accessible at http://genome.lbl.gov/vista/ ), using the mVISTA option as appropriate for aligning and comparing genomic sequences from multiple species. Within mVISTA, 'Shuffle-LAGAN' [64] was chosen as the alignment program, no repeat masking was performed, and "translated anchoring" was selected for its reported potential to improve alignment of distant homologs. VISTA was run twice, with two different "reference sequences" that the alignment is anchored to: genomic periostin sequence from chicken was used as reference sequence to study exon 21V22 (Additional file 2, Figure S1 and Additional file 3, Table S2A), and genomic periostin sequence from human was used to study exon 17 (Additional file 3, Table S2B).

### Sequence logos

Sequence logos were generated using the internet-based tool WebLogo (version 2.8.2) [65] (accessible at http://weblogo.berkeley.edu/), without small sample correction and with a customized color scheme.

For tetrapod exon sequence logos, exon-specific alignment blocks from seven tetrapod species (Additional file 1, Table S1) were submitted individually for sequence logo generation. The teleost repeat sequence logo was obtained by manually aligning the individual 13 amino acid repeat units within exon 18=19 from *D. rerio*, locus 1 (6 repeat units) and locus 2 (13 repeat units), *G. aculeatus*, locus 1 (7 repeat units) and locus 2 (5 repeat units), *T. nigroviridis*, locus 2 (8 repeat units), and *T. rubripes*, locus 2 (6 repeat units), totaling 45 repeat units. Alignment involved the occasional introduction of gaps and, in one case, the deletion of one amino acid from one repeat sequence to avoid the introduction of a gap position into the repeat alignment. This repeat alignment block was then duplicated (side-by-side) before being submitted for sequence logo generation.

### Repeat visualization with dot-matrix plots

To visualize repetitive patterns within sequences with 2-dimensional matrix dot plots, the JAVA application JDotter [66] (accessible at http://athena.bioc.uvic.ca/tools/JDotter) was used. For genomic DNA sequences, (Additional file 4, Figure S2) we used a sliding window of size 50 (default) and a DNA scoring matrix of +5 for a match and -4 for a mismatch. Dot plots were originally generated at a resolution of 1 nucleotide per pixel and displayed with the following parameters for the "GreyMap Tool" to optimize visibility of the repeats in the display: 0 (top)/35 (bottom).

For protein sequences, we used a sliding window of size 5 and the amino acid scoring matrix BLOSUM62. The dot plot was generated at a resolution of 1 residue per pixel and with default "GreyMap Tool" parameters 0 (top)/245 (bottom) for the matrix display to obtain the maximum dynamic range of grey-values. The grey value continuum underlying this matrix display was then converted into a color continuum (white-yellow-orange-red-black) using standard image manipulation software.

### Secondary structure predictions

For secondary structure predictions, we used PsiPred [67] implemented as a web-based tool [68] (accessible at http://bioinf.cs.ucl.ac.uk/psipred/). PsiPred v2.5 was used for all predictions. Predictions were performed without any filtering options selected, in particular without the option to mask low complexity regions (selected by default).

### Determination of periostin exon 21 and exon 21V22 in Xenopus laevis

For 21 EST sequences from *X. laevis *periostin, the UCSC browser indicated additional sequence without genomic match at a position coincident with an assembly gap between scaffold_505_18 and scaffold_505_19. The EST subsequences without genomic coverage were analyzed and determined to be identical, with a length of 81 nucleotides. Protein sequence comparisons with chicken periostin showed that the unmatched subsequence is periostin exon 21 and that the following exon (seen in 9 out of a total of 33 EST sequences covering this region) is actually exon 21V22 (data not shown).

## Results

### Exon naming conventions

Periostin is universally described as a 23-exon gene. Our findings, going beyond the scope of human and mouse as the organisms in which periostin has been investigated, expand and modify this notion on multiple counts. To keep our exon designations consistent with those in the existing literature while maintaining the capability to capture the new findings presented here, we decided on the following rules:

We consider exons 1 - 23 as described for human and murine periostin "canonical" and leave their numbering unaltered. We refer to homologous exons in other species by the same numbers, even if variations in exon count were found to exist.

Additional or modified exons without direct counterparts in human or mouse are referred to by special designations we are introducing here (see also Additional file 1, Table S1).

### The tetrapod periostin C-terminal region is homologous to a repetitive part of teleost fish periostin

Our early attempts to identify homologs of known function to the C-terminal part of human or murine periostin using sequence similarity searches in protein databases produced exclusively hits to corresponding regions in tetrapod periostin sequences. An iterative search (PSI-BLAST) with this region (exons 16 - 23) alone produced an alignment (data not shown), with a sequence fragment of zebrafish (*Danio rerio*) periostin marked by a highly conserved 5-fold 13-amino acid repeat [39], covering exons 16 - 20 of the human periostin query.

In order to ascertain actual homology between the obviously repetitive part of zebrafish periostin and the C-terminal part of mammalian periostin, we conducted a profile-based homology search with repeat units of the zebrafish periostin (see Methods for details). We found that a search with only one repeat unit resulted in spurious hits, i.e., protein sequences of generically repetitive nature, e.g., myosin. However, using as few as two repeat units together for the search produced as the top hits specifically periostin C-terminal sequences from other vertebrate organisms, including human and mouse.

These results confirmed an evolutionary relationship between mammalian periostin C-terminal sequence and a zebrafish periostin repeat region, but since no significant hits to other proteins were found, it also marked the "end of the road" for attempts to identify similarities to gene sequences other than periostin for a tentative functional annotation. We hence decided to systematically compare complete periostin sequences and especially their C-terminal region across a range of phylogenetically diverse vertebrates in the hope of gaining clues to the functional role of the C-terminal region.

### Full length periostin sequence, including the alternatively spliced part, can be inferred for many vertebrates with genomic data

Besides human and mouse as representatives of placental (eutherian) mammals, we selected as the basis for detailed study of periostin the complete genome sequences of the following tetrapod species: gray short-tailed opossum (*Monodelphis domestica*), platypus (*Ornithorhynchus anatinus*), chicken (*Gallus gallus*), green anole lizard (*Anolis carolensis*), and western clawed frog (*Xenopus tropicalis*). Furthermore, teleost fish genomes were added, namely of zebrafish (*Danio rerio*), stickleback (*Gasterosteus aculeatus*), medaka (*Oryzias latipes*), and two pufferfish, *Tetraodon nigroviridis *and *Takifugu rubripes*, adding up to 12 vertebrate - or, more narrowly, euteleostome - species.

Full-length periostin sequences comprising all exons are only readily available or annotated in public sequence databases for a subset of these vertebrate species. This was the case for human, mouse, chicken, frog, and a subset of teleost fish, although for some of these, several mRNA and/or EST sequences had to be combined to obtain complete sequences, or quality deficiencies in the genomic assembly required additional efforts (in *X. tropicalis*). For the remaining species, the periostin coding sequence was inferred using a combination of approaches (detailed in Methods). A detailed account of the origin and status of each species' periostin protein sequence is provided in Additional file 1, Table S1, and the actual sequences are provided in a comprehensive listing in Additional file 5. In the same table, we summarize existing evidence for periostin transcript variation and for other unusual features of periostin gene structure in the 12 species selected for detailed analysis.

We will return to a detailed analysis of the 13 amino acid repeat motif later in the manuscript and focus first on transcriptional and genomic evidence for periostin isoforms.

### Alternative splicing events specific to the periostin C-terminal region are observed in all euteleostome lines with sufficient transcript evidence, including teleost fish

Alternative splicing affecting the region from exon 17 to exon 21 has been described for human [1] and murine periostin [2]. By evaluating available transcript evidence for the vertebrate species considered in this work, we universally found evidence for alternative splicing specific to this region for all species with non-trivial amounts of sequence data (see Additional file 1, Table S1 for details). This includes three fish species (zebrafish, stickleback, and medaka). Note that Additional file 1, Table S1 lists also transcript evidence for alternative periostin 5' and 3' ends observed in some species, including human.

Available periostin transcript evidence suggests that the set of exons subject to alternative splicing is also species-dependent (Additional file 1, Table S1). This observation is subject to considerable uncertainty due to limited transcript coverage, but human and murine periostin are compelling examples, having substantial amounts of transcript evidence. We found that in human, exons 17, 18, 19, and 21 - but never 20 - are alternatively spliced, but in mouse, alternative splicing does affect exon 20, as well as exons 17 and 21.

In summary, the aggregation of transcript data suggests that the occurrence of alternative splicing is a universal hallmark of this part of the periostin gene. This transcriptional variability could be important for a complete understanding of periostin function.

### A 24th periostin exon is functional, expressed, and alternatively spliced in some tetrapod lines

Both human and murine periostin have been characterized as having a maximum of 23 exons ("canonical exons"), according to a substantial amount of periostin mRNA and EST sequences in these organisms.

Our analysis of the chicken periostin locus revealed the existence of an additional non-canonical exon situated between canonical exons 21 and 22, which we termed "21V22" to reflect this property. Exon 21V22 is present in 8 of the 15 mRNA/EST sequences covering this region. It is flanked by canonical splice sites and is 87 nucleotides long, allowing for maintenance of the reading frame without stop codon and thus translation into protein.

Sequence similarity searches with the protein sequence of this exon against genomic sequences produced clear matches in zebra finch (*Taeniopygia guttata*), lizard, opossum, and platypus, which are devoid of periostin transcript evidence. In all of these, the most basic hallmarks of a functional exon (maintenance of the reading frame without stop codon, canonical flanking splice sites) were also found, suggesting that this exon may also be functional in these species. However, equivalent searches failed to produce hits for this exon in frog (where we identified it separately, see below) and, interestingly, in placental mammals (human, mouse, dog, cow). While this echoes EST evidence for these species, we were surprised by the complete absence of even low quality hits in the genomic sequences, and performed a multiple alignment of periostin locus genomic sequence from 33 vertebrate species to get a clearer understanding of the "fate" of this exon across vertebrate taxa.

The genomic sequence alignment (Additional file 2, Figure S1) with chicken periostin as a reference shows a clear conservation peak for exon 21V22 not only for the aforementioned species for which this exon is known or highly likely to be expressed (chicken, finch, lizard, opossum, platypus; >75% conservation), but also for most other mammals tested (<75% conservation), including human.

We then analyzed the nucleotide sequences underlying the exon 21V22 conservation peaks (Additional file 3, Table S2A). We noted that for those species where the conservation peak was below 75%, hallmarks for a functional exon in general (maintenance of reading frame, no stop codons, flanking intronic GT-AG splice sites) were missing in many cases. In some others, these hallmarks are preserved, and the exon could be functional.

For example, in human, there could theoretically be exon 21V22 expression within full-length periostin transcripts and translation into protein product, as underscored by the GENSCAN [69] gene model NT_024524.504 (UCSC browser, Human Mar. 2006 Assembly), with a terminal exon identical to exon 21V22. The fact that it has never been experimentally observed leads us to speculate that the encoded protein sequence may either be expressed in unobserved conditions or be incompatible with human periostin function and remain unexpressed.

We conclude that exon 21V22 is a periostin exon not previously described. It is located between exons 21 and 22, and related in sequence to exons 17 - 21. Like these, it is subject to alternative splicing. Its expression is confined to a subgroup of vertebrates, including birds, where its expression is *de facto *observed in chicken, amphibians (see below), and putatively in lizards and non-placental mammals.

### X. tropicalis expresses periostin exon 21V22 and a cluster of up to 8 additional genomically encoded exon 19 copies

**Figure 2:**
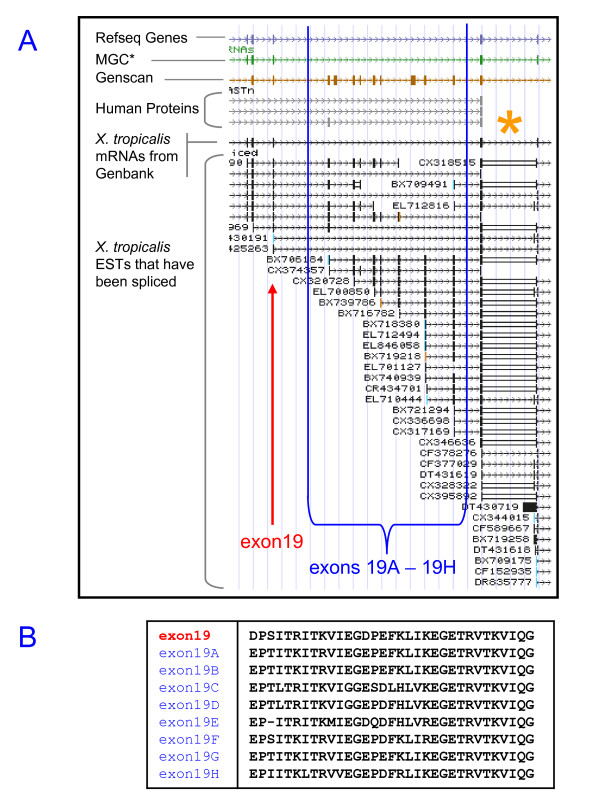
**The *Xenopus tropicalis *periostin C-terminal region in the UCSC genome browser**. **(A) **The 'Spliced EST' track shows a cluster of eight additional genomic copies of exon 19 (referred to here as exons 19A - 19H) immediately downstream of exon 19. These exons, not represented in the RefSeq record, are subject to alternative splicing in various combinations. The asterisk (*) marks a region where multiple ESTs have subsequences unaligned to genomic sequence due to a gap in the assembly (not shown), which we resolved to be exon 21 (see main text). **(B) **Amino acid sequences of exons 19 and 19A - 19H. The very high degree of similarity among these is evident.

When examined in the UCSC genome browser, the periostin locus of the claw frog *Xenopus tropicalis *presents a notable feature: the number of (alternatively spliced) exons upstream of exons 21 and 21V22 by far exceeds the number of exons encountered in periostin from any other tetrapod species. Specifically, compared to mRNA sequence BC154911.1 as a reference, EST sequences indicate up to eight additional exons between exons 19 and 20. Analysis of these eight exon sequences revealed that they are extremely similar to *X. tropicalis *exon 19 (Figure 2), and we refer to these exons as exons 19A to exon19H. We are confident that these exons are not an artifact due to the preliminary nature of the *X. tropicalis *genome assembly, because they are present in both genomic and transcript sequence. Furthermore, we found a similar cluster of at least seven exon 19-like sequences among *Xenopus laevis *transcript sequences via similarity searches with *X. tropicalis *exons 19 and 19A - 19H as a query sequence (e.g. *X. laevis *mRNA GenBank:CB200763.2; data not shown).

An overview of all *X. tropicalis *ESTs covering the exon 19/20 region is given in Additional file 3, Table S3. Comparing the library origin between ESTs splicing directly from exon 19 to exon 20 (5 ESTs) to those that contain at least a subset of exons 19A - 19H (24 ESTs) revealed a strong bias for embryonic or metamorphic origin in the latter set. While far from definitive, this suggests the possibility of a developmental role of exons from the 19A - 19H cluster.

We furthermore analyzed the region without genomic match occurring in a majority of ESTs downstream of the exon 19 cluster (marked '*' in Figure 2), co-incident with a gap in the genomic assembly, and concluded that the unmatched region is periostin exon 21 and that the following exon (seen in 9 out of 33 transcript sequences covering this region) is actually exon 21V22 (see Methods).

### The predicted basic secondary structure of the periostin C-terminus, consecutive beta sheets separated by turns, is conserved in periostin sequences across vertebrate species regardless of exon structure

**Figure 3:**
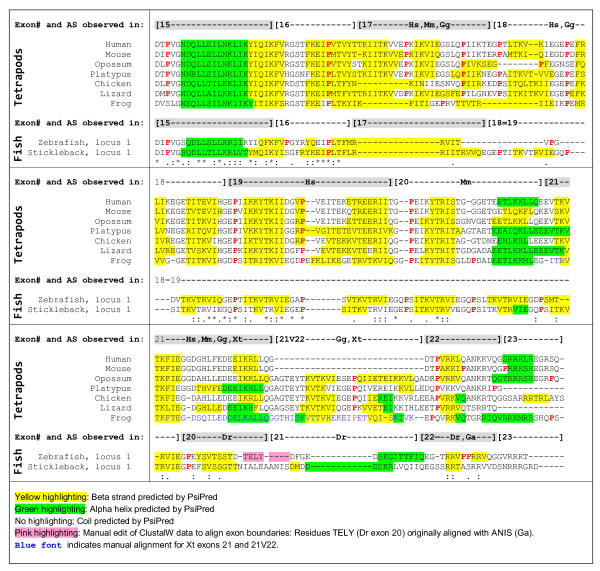
**Multiple sequence alignment and secondary structure prediction results for the periostin C-terminal region**. A ClustalW alignment for periostin's C-terminal region of seven tetrapods (Hs, Mm, Md, Oa, Gg, Ac, Xt) and two teleosts (Dr locus 1, Ga locus 1) is shown. The exon structure is indicated above the alignment, separately for tetrapods and teleosts, together with two-letter species codes, if alternative splicing is observed for a particular exon in that species. Residues are highlighted based on PsiPred secondary structure predictions performed separately for all nine sequences as indicated below the alignment. The general alignment of the secondary structure elements, mostly beta strands, across all species considered here, is obvious.

We subjected periostin protein sequences exons 15 to 23 from 7 tetrapods (human, mouse, opossum, platypus, chicken, lizard, and frog) and 2 teleost fish species (zebrafish, locus 1 and stickleback, locus 1, see below) to a secondary structure prediction algorithm (See Methods for details). Prediction results are shown in Figure 3 in the context of a multiple sequence alignment of the nine sequences.

Generally, results show a universal multi-beta strand structure starting at the end of exon 15 and continuing into exon 22. Starting in exon 17, beta strands are usually separated by short coil stretches around proline residues occurring with a periodicity of 13 (or close to 13) in fish and at often somewhat larger intervals in tetrapods. (Note that for practical reasons, we are referring to the exon following exon 17 as exon "18=19", because for many of the teleost periostin sequences, it allows us to keep a numbering scheme on the basis of 23 periostin exons intact).

Evaluating the alignment and the secondary structure predictions in more detail, the following exceptions and refinements are notable:

-	For tetrapod exons 17 to 21 (and 21V22, where applicable), each exon accommodates two beta-strands.

-	Starting with exon 20 (middle, Xt: exon 21), the recurring proline residue is "lost" in tetrapod exons 20 and 21 and "reappears" universally in exon 22. It is also present in exon 21V22 (middle) found in only five of the seven species considered here.

-	For the most part, the pattern of beta strands separated by coils continues through exons 20 and 21 despite the absence of a proline residue. However, interestingly, exon 20 (second half), and exon 21 (second half) show a predicted alpha helix instead of a beta sheet in some of the species.

-	Due to the strongly conserved repeat structure in teleost fish, the beta strands are very regular here. Remarkably, they are also in phase with the tetrapod beta strands in the multiple sequence alignment.

-	In the multiple sequence alignment, teleost exon 21 is aligned with tetrapod exon 21V22 and teleost exon 20 is aligned with tetrapod exon 21 (partial). This reflects both the length of teleost exon 18=19 accommodating, in the alignment, multiple tetrapod exons, and the uncertainty regarding a direct orthology between the short teleost exons 20 and 21 and tetrapod exons 20 and 21.

The predicted structure of two beta-strands per exon is interesting in light of the transcriptional variability these exons are subject to: Evidence for alternative splicing exists for each of the exons from 17 to 21V22 in at least one tetrapod species (Additional file 1, Table S1). The genomic duplications of exon 19 in *Xenopus tropicalis *are also alternatively spliced (Additional file 3, Table S3). Alternative in- or exclusion of any of these exons will then add or remove pairs of beta-strands to or from the protein.

### Teleost fish genomes have two copies of periostin

Identifying the genomic periostin locus was straightforward in all tetrapod genomes, even in those lacking periostin transcript data (lizard, platypus, opossum) based on relevant multi- or cross-species tracks in the UCSC Genome Browser.

However, it is believed that the common ancestor of teleost fish has undergone whole-genome duplication (WGD) or at least a large-scale gene duplication event [70], complicating the situation. A systematic genome-wide sequence search (BLAT) with human periostin sequence against the five fish genomes resulted in typically three distinct matches. Analysis of these matches in terms of length and similarity indicated that the two stronger matches corresponded to periostin. This was confirmed by the reciprocal results of a genomic search with human TGFBI sequence. Here, the best match was always the third-ranked hit from the previous periostin genomic search (data not shown).

We conclude that teleost fish retained both copies of periostin after the duplication event. By contrast, the second copy of TGFBI was either universally lost (i.e. presumably before the teleost species radiation), or TGFBI was not part of the duplicated gene complement. Our designation of the two periostin loci in the fish species as "locus 1" and "locus 2" (see Additional file 1, Table S1 for genomic coordinates) was done on the basis of expression level as judged by the amount of transcript evidence mapped to either locus in the UCSC genome browser. Where possible (zebrafish and tentatively stickleback), we assigned the label "locus 1" to the locus found to be more highly expressed by that measure, coinciding in the case of zebrafish with the version of periostin described in the literature [21]. For the other fish species, we based the assignment on the grouping resulting from a multiple alignment-based dendrogram of teleost periostin protein sequences (see below).

We reconstructed as completely as possible periostin coding sequence from both loci for four of the five teleost fish species, relying mostly on sequence similarity searches with the known periostin sequence from zebrafish. In several cases, missing exons or other uncertainties remain due to gaps in genomic sequence and/or complete lack of any guiding transcript coverage. For one species (medaka), reconstruction from one periostin locus was not undertaken due to the fragmentary nature of the genomic sequence data.

**Figure 4:**
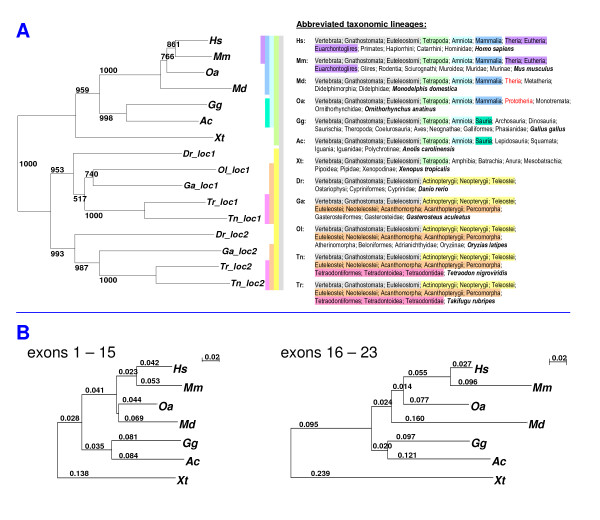
**Phylogenetic trees based on alignments of periostin sequences**. (A) The phylogenetic tree obtained from a protein sequence alignment of periostin from seven tetrapod species (Hs, Mm, Md, Oa, Gg, Ac, Xt) and 5 teleost species (Dr, Ga, Ol, Tr, Tn). Numbers at the branch points are bootstrap values. For the teleosts, both periostin loci were considered, except for Ol, where periostin from the second locus was not reconstructed. The dendrogram accurately reproduces established taxonomic lineages (adapted from the NCBI Taxonomy Browser [85]) for the species in question as given on the right, with the minor exception of the branching order of Md and Oa. It clearly places the periostin duplication event at the base of the teleosts, with the same subtree structure replicated between the two loci. (B) Phylogenetic trees based on tetrapod protein sequence alignments of periostin exons 1 - 15 (left) and 16 - 23 (right), scaled for identity of branch length units (see 0.02 marker), which are given at the branch points. The right dendrogram has generally longer branches, most profoundly for Xt, reflecting the increased rate of evolution of periostin's C-terminal region.

A dendrogram based on an alignment of seven tetrapod periostin sequences (see above) together with the nine teleost periostin sequences shows, among the teleosts, a separation into two major branches by periostin locus 1 vs. 2 (Figure 4A) preceding the branching into the multiple teleost lineages, and thus echoing the genome duplication event. Within each branch (i.e., periostin locus), the dendrogram structure recapitulates the phylogenetic relationship between the five teleost fish species (minus medaka for one locus), i.e., zebrafish as a member of the Ostariophysi lineage most distant from the other four species (Euteleostei), and the two puffers (Tetraodontidae) Takifugu and Tetraodon closest together.

### Periostin sequence conservation is dramatically lower in its C-terminal region

Up to exon 16, periostin sequences are extremely well conserved between all euteleostomes, including the two periostin loci in teleosts. In fact, not counting exons not reconstructed due to missing genomic sequence and uncertainties around the boundaries of Ensembl-prediction based exons 15 and 16 for locus 2 in Tetraodon, a multiple sequence alignment for all seven tetrapod and nine teleost periostin sequences showed only 5 gapped positions from exon 2 through 16.

By contrast, for tetrapod periostin sequences alone, and not counting gaps corresponding to entire exons (see below), we counted 32 gapped columns in a multiple sequence alignment from exons 16 to 22. This increased speed at which the C-terminal part of periostin evolves compared to the larger N-terminal portion comprising the EMI and fasciclin domains is also reflected in alignment-based dendrograms derived separately for these two sections of periostin sequence (Figure 4B). The overall tree topology is identical in both cases (with the exception of the relative positioning of opossum and platypus), but branch lengths are markedly longer for the C-terminal region-based alignment. Finally, further elements of genomic and transcriptional variation, discussed in detail below and exclusively found in the C-terminal periostin region, are adding to the picture of a part of the periostin gene that shows highly divergent and dynamic characteristics.

Starting with exon 17, we found in some cases dramatic variation with respect to exon number, exon length, and overall sequence conservation, some of which we described above for tetrapod species. The divergence for this region is, in some respects, even higher between teleost species.

In fish, we observed exon 17 to vary in length between 5 and 11 amino acids. Interestingly, short versions of exon 17 also occur among tetrapod periostin sequences, where the standard exon 17 length is 26 amino acids: birds (chicken and zebra finch) as well as frog (but not reptiles) have an exon 17 that is about 16 amino acids in length.

Exon 18=19 is especially remarkable in teleost fish: in periostin sequences from all species except medaka (*Oryzias latipes*), we found it to be longer than neighboring exons, sometimes remarkably so - for stickleback periostin from locus 2, it is 91 amino acids long (with EST coverage); for zebrafish locus 2, it appears to encode 187 amino acids (incomplete EST coverage). This exon is marked by a repeat structure that is obvious to unaided visual inspection and readily surfaced via high-quality secondary hits in genome-wide sequence searches with nucleotide or protein sequences containing the repeat. It was chiefly this exon (in zebrafish) which we had initially observed aligning to the human periostin query of exons 16 - 23 in a iterative sequence search, and it is this exon from which the repeat sequence for the profile-based homology search was taken.

### Characterization of the periostin repeats

**Figure 5:**
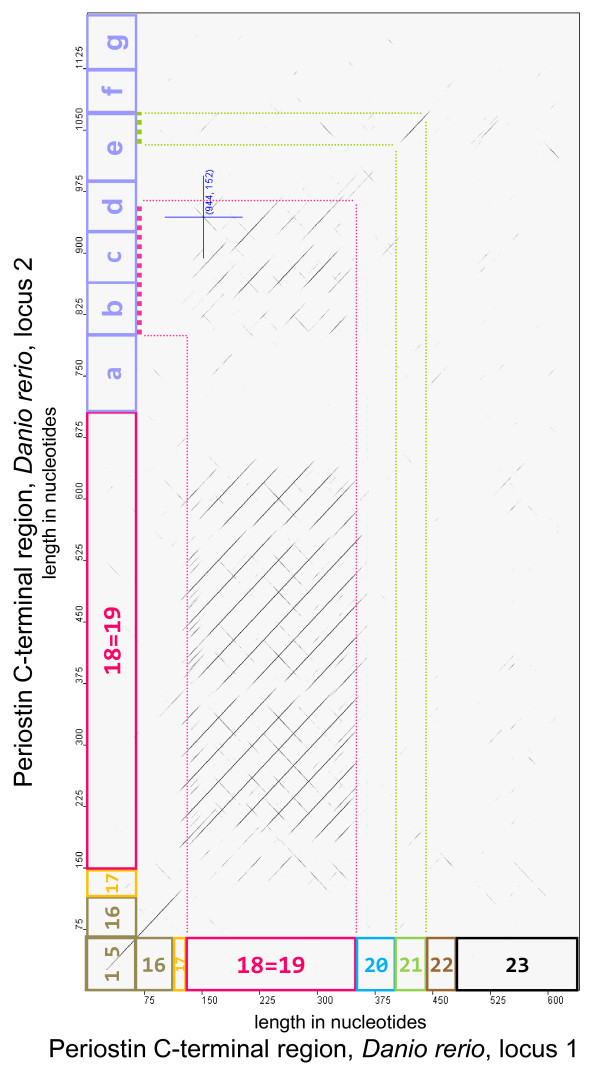
**Matrix dot plot of periostin nucleotide sequences from the C-terminal region of zebrafish locus 1 (horizontal) versus locus 2 (vertical)**. Exon sizes and boundaries are indicated in either dimension, with locus 2 exons downstream of 18=19 marked here provisionally as a - g. The dot plot shows the 39 nucleotide repeat pattern within exon 18=19 and also reveals similarities to the repeat within locus 2 exons b, c, d as well as a correspondence (probable homology) of locus 1 exon 21 to locus 2 exon e (partial). Weaker reverse-complement similarities are also visible.

The periodicity of this repeat is generally 39 nucleotides/13 amino acids. Slight deviations from this length are observed in a fashion that keeps the reading frame intact. We used matrix dot plots to get a more detailed understanding of the repeat properties (see Methods for details). Interestingly, the repeat exhibits similarity to its reverse complement to varying degrees. The most notable example here is in stickleback periostin locus 1, where exon 18=19 reverse-complement similarity reaches 25/39 (64%) nucleotide identities with no gaps. This level of similarity is readily revealed in the matrix dot plots as lines orthogonal to the main diagonal. Other repeat instances show weaker levels of reverse-complement similarity that are not readily surfaced in the dot plots (e.g., 19/42 identities including a gap of 3 positions for zebrafish periostin exon 18=19 at locus 2 (Figure 5)).

Visualizing genomic periostin sequence extending beyond the boundaries of the repeat exon(s) in dot-matrix plots, we observed that the repeat pattern appears confined to the exons and does not extend into adjacent intronic sequence (Additional file 4, Figure S2).

The matrix dot plot in Figure 5 further illustrates our finding that the number of exons beyond exon "18=19" in teleosts is variable: Zebrafish locus 1 periostin has 4 more exons, but locus 2 periostin has as many as seven, the largest number we found. Many of these "extra" exons also show the repeat structure observed in exon 18=19 (Figure 5). On the other hand, periostin from the stickleback locus 2 has only 3 more exons after exon 18=19, one less than the canonical number.

**Figure 6:**
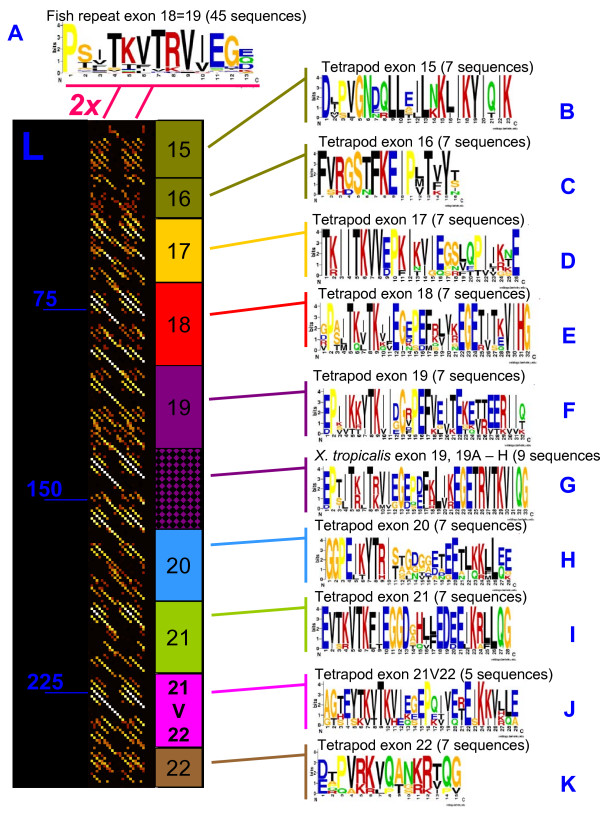
**Sequence logo representations of the teleost fish periostin repeat consensus and of the consensus sequences from tetrapod exons of the periostin C-terminal region**. **(A) **The periostin repeat sequence logo for teleosts was obtained from an alignment of the 13 aa repeat units of periostin exon 18=19 (see main text) from four teleost species (Dr, Ga, Tn, Tr). The starting amino acid (proline) is arbitrarily chosen (see also Methods). **(B - K) **The exon-specific sequence logo representations were obtained from alignments of the respective periostin exons from seven tetrapod species considered here (Hs, Mm, Md, Oa, Gg, Ac, Xt). Exceptions are (G), the Xt exon 19 cluster, where a sequence alignment of exons 19 and 19A - 19H (see main text) was the basis for the sequence logo, and (J), exon 21V22, which was based only on sequences from Md, Oa, Gg, Ac, and Xt. **(L) **A matrix dot plot showing similarities between the teleost repeat consensus (horizontal, as duplet for clarity) and the sequential tetrapod exon consensus sequences (vertical), with the exon sizes indicated on the side. The dot plot is color-coded (white-yellow-orange-red-black), with white indicating highest similarity and black indicating no similarity. Both this dot plot and the sequence logos indicate the strong similarities between the teleost repeats and the tetrapod C-terminal exons found in the alternatively spliced exons 17 - 21V22 (see main text).

On the protein level, the 39-nucleotide repeats encode a 13-amino acid sequence with the following consensus: ***PS***I***TK***V***TRV***I***EG***E. (Figure 6A; amino acids that in italics were found to be the most frequent at this position in every locus-specific repeat alignment considered, which were for both loci from zebrafish and stickleback, and for one locus from each of the two puffers, *T. nigroviridis *and *T. rubripes*). Its key characteristics as given here are: (i) a Pro at position 1, (ii) a stretch of hydrophobic amino acids (mostly Thr and Val) from positions 3 to 10, interspersed with the positively charged amino acids Lys and Arg in positions 5 and 8, and (iii) a final stretch of three amino acids that is dominated by negatively charged Glu and Asp (mostly at positions 11 and 13, with a Gly in between).

Tetrapod periostin sequences do not have a large exon with obvious repeats like most teleost fish. However, dot matrix plots readily reveal self similarities within the transcript region spanned by exons 17 - 22 (Additional file 4, Figure S3).

Similarities to the teleost repeat are likewise demonstrated: Figure 6B-K gives sequence logo representations [65] for the teleost repeat described above and for periostin exons 15 - 23 derived from the seven tetrapods considered here (Hs, Mm, Md, Oa, Gg, Ac, Xt). A color-coded version of a matrix dot plot is shown, generated by comparing 2 instances of the teleost repeat consensus (26 aa) against concatenated tetrapod consensus sequences from exons 15 - 23. The highest similarities to the 13 aa repeat are detected in exon 18, exon 19 (*X. tropicalis*, see below), exon 21, and exon 21V22 (see below), whereas we see somewhat weaker similarities in exons 17, 19, and 20. Similarities in exons 15, 16, and 22 are markedly weaker than those found in the exons in-between.

The concept that tetrapod exons between 16 and 22 are homologous to the long repeat exon 18=19 in teleost fish is powerfully reinforced by the alignment given in Figure 3. This alignment shows not only the primary structure (sequence), but also the predicted secondary structure elements. Here, tetrapod exons 17 to 21V22 align with the teleost repeats such that the sequence alignment results also in an alignment of the secondary structure elements, i.e., the beta strands. Critically, tetrapod exon 21V22, which appears downstream of exon 21, shows an especially high similarity with the teleost repeat consensus: for example, the subsequence EYTKVTKVIEGEP from chicken exon 21V22 is 77% identical to the teleost repeat consensus ***S***I***TK***V***TRV***I***EG***E***P ***(rearranged here for a terminal P to align with the chicken sequence). Thus, exon 21V22 defines the range of tetrapod exons homologous to the long teleost repeat exon.

## Discussion

### The C-terminal region of periostin is likely a key to disambiguating periostin function

Despite ongoing discussions regarding the periostin alternatively spliced C-terminal region (e.g. [42,71]) dating back to its original description [1,2], this region has not been target of in-depth analyses to identify its biological function. Here, we presented evidence for its universally high degree of variability in a phylogenetic context, which we hope will assist future research on periostin. We laid out the multiplicity and, within the euteleostomes, phylogenetic universality of modalities observed (including alternative splicing as well as genomically encoded variable exon counts and lengths) to modify this part of periostin. Considering also that the functionally distinct periostin paralog TGFBI lacks an equivalent to periostin's C-terminal region, we propose that this region is of functional relevance, and that the sequence variation imparted by alternative splicing could be a modulator of periostin function.

In studies aimed at elucidating determinants of tumor-suppressive properties of periostin, the periostin C-terminal region was found to be sufficient to suppress anchorage-independent growth in T24 bladder cancer cells [72], to suppress cell invasiveness in SBT31A bladder cancer cells, and to abrogate the metastatic potential of highly metastatic B16F10 mouse melanoma cells in an *in vivo *assay [42]. While the tumor-suppressive properties documented for periostin in these studies remain to be reconciled with a large body of literature describing periostin as tumor promoting, these results demonstrate the functional significance of the periostin C-terminal region as a whole. This notion is also playing out in discussions in literature not explicitly concerned with decoding periostin function on the sequence or domain level. For example, the significance of a recent high-profile study on periostin as a potential therapeutic target after heart attack [73] has been questioned on the basis of this work being partly based on a version of periostin lacking the C-terminal region [13,25,74].

The situation is more complex when it comes to different periostin splicing isoforms characterized by individual presence or absence of cassette exons 17 - 21 (human and mouse). For the following discussion, we are using a notation of exon numbers preceded with a minus sign in superscript to characterize periostin isoforms by their absent exons relative to a full-length periostin, denoted POSTN^fl^, with a full complement of exons, i.e. 23 for human and mouse.

Based on the common occurrence of gel pictures showing multiple periostin bands throughout the literature [e.g. 2,4,15,17,25,29,75,76], we can assume that studies on native periostin typically deal with a mixture of isoforms, although the exact number is usually unknown. In apparent agreement with the number of four isoforms originally reported [2], most gels show a cluster of 4 or sometimes 5 periostin bands. However, the similar length of the alternatively spliced exons gives rise to possible combinations that are very close in size, so that the actual number is potentially higher due to isoforms co-migrating in the same band. Indeed, studies have found higher isoform counts, for example 6 in human kidney cancer [76] and more than 8 in human breast tissue (Hoersch et al: Alternative splicing of periostin in human breast cancer, manuscript in preparation), and in both cases, gel pictures allowed distinction of only four or five variants.

Descriptions of experimental procedures manipulating periostin in some way, for example expressing in ectopically in a cell line, are not always explicit on the specific isoform used, and the attributes "full-length" or "wild type" are not always or unambiguously referring to the isoform with all exons present. In some instances, the relevant isoform can be traced via database accession numbers provided, but overall, this information is not consistently enough available to enable a meaningful isoform-specific meta-analysis.

More interestingly, biological observations regarding periostin have, in some cases, been explicitly tied to specific variants, as illustrated by the following three examples.

(i) In a study on periostin expression in bone [71], the designation "periostin-like factor" or "PLF" was introduced by the authors specifically for periostin isoform POSTN^-21^, while the designation "periostin" refers to isoform POSTN^-17 ^in the same study. Using the same designations, this group subsequently described differential spatiotemporal expression patterns of these two isoforms in the developing mouse embryo [77], although it has to be understood that the "isoform-specific antibodies" used in this work are technically only site-specific for exons 21 and 17, respectively, and could, in principle, report on multiple isoforms characterized by exon 17 or exon 21 presence.

(ii) Differential expression profiles over time were observed for different periostin isoforms after a myocardial infarct event in periostin-null mice, with isoform POSTN^-17-21 ^being expressed strongest initially and then becoming weaker after a sustained period of high expression as the expression level of other isoforms gradually increased [14].

(iii) In a continuation of earlier work, it was found that periostin isoforms POSTN^fl ^and POSTN^-17-21 ^abrogate invasion in cell lines B16F10 and SBT991 and *in vivo *lung metastasis of B16F10 cells in mice, while isoform POSTN^-17-18-21 ^does not [51]. Along similar lines, but with opposite directionality, isoform-specific differences in the ability to promote migration and invasion were observed in breast cancer cells (Hoersch et al: Alternative splicing of periostin in human breast cancer, manuscript in preparation).

Apart from the often implicit issue of periostin isoform expression, several aspects of periostin are controversially described and discussed in the literature to date, prominently among them: (i) periostin subcellular localization, (ii) the cell types responsible for periostin expression and the physiological sites of periostin protein localization post secretion, and (iii), in the cancer literature, periostin function being tumor promoting or suppressing.

It will be interesting to see to what extent the variability of periostin isoform expression will contribute to resolving these issues, a possibility that is commonly raised in discussion sections in the periostin literature. Given the sequence and predicted structural similarities between periostin's cassette exons due to the underlying repeat structure laid out in this study, we detail below a functional interpretation that employs a dose-type rationale, i.e. a binding interaction as a function effectively tuned by exon count. This seems more compelling to us than a purely exon-specific mechanism, although the reality may well be a complex overlay of both mechanisms.

### The periostin C-terminal region exhibits exceptional transcriptional and genomic variability

We demonstrated the homology of periostin's multi-exon C-terminal region in tetrapods and its C-terminal region in teleosts, where a 13 amino acid sequence is stacked into adjacent repeats. The majority of these is, in most of the teleost species studied, concentrated in one exon that is unusually large (dubbed "exon 18=19" in this work).

The absence of protein domain signatures in periostin's C-terminal region is thus no longer puzzling. We can assume that this region as observed today evolved from an ancestor repeat region, leading to its distribution over multiple exons, particularly in tetrapod species.

We demonstrated that the periostin C-terminal region is showing increased evolutionary plasticity relative to the remainder of the gene. This is evident from comparing branch lengths of phylogenetic trees based on separate sequence alignments of the C-terminal region and of the remainder of the gene (Figure 4B). Moreover, it is reinforced by the variability we found almost exclusively for periostin's C-terminal regions between different taxa that are not even reflected in these dendrograms: profoundly different exon structure (teleosts vs. tetrapods), marked divergence between the two periostin copies in teleosts, variable exon length (exon 17 across euteleostomes, exon 18=19 in teleosts), an additional exon not previously described (exon 21V22 in tetrapods except placental mammals), and additional genomic copies of exon 19 with a strong developmental implication (in frogs).

We also reported that alternative splicing is a universal hallmark of the periostin C-terminal region. While long established for human and murine periostin, we found evidence for alternative splicing events affecting multiple exons in periostin's C-terminal regions in all organisms studied here (including teleost fish) for which non-trivial amounts of transcript sequence data were publicly available. Exons 17, 18, 19, 20, 21, and 21V22 are all alternatively spliced cassette exons, although it appears that in any given species, at least one of them is constitutively expressed. Although uncertainties remain in some cases due to low sequence coverage, the predicted alternative splicing patterns were found to vary by species, sometimes subtly so (e.g. in human and mouse with regard to exon 20).

Taken together, these observations point to periostin's C-terminal region being exceptionally dynamic on different scales. On a transcriptional scale, alternative splicing could give rise to periostin variants in a tissue, development, or disease process dependent manner, the functional impact of which is only beginning to emerge. On a genomic, i.e., evolutionary scale, the increased plasticity of this region leads to periostin configurations that are specific to taxonomic lineages or groups.

### The periostin C-terminal region and neofunctionalization

We understand this increased evolutionary plasticity as a manifestation of neofunctionalization [78] affecting euteleostome periostin where we observe variations in exon length, count, structure, and variable splicing patterns in the C-terminal region. We hypothesize that the repeats were acquired as *de novo *sequence in an exonization event [79], either by ancestral periostin (after the periostin/TGFBI split) or by the common periostin/TGFBI ancestor gene, in which case the repeats were lost by TGFBI after the split.

Data available at this time is insufficient to distinguish between these two possibilities, chiefly because of the preliminary nature of relevant genome assemblies outside the euteleostomes (for details and Figures, see Additional file 6). This newly exonized repeat sequence was then relatively free to evolve, while the remainder of the gene remained much more conserved due to the structural and functional constraints imposed by the EMI and FAS1 domains.

Remarkably, our observations concerning the two periostin copies in teleost fish point to the same mechanism of neofunctionalization playing out here. Between the two periostin copies in teleosts, we are again seeing variations in exon length, count, and structure. In addition, to the extent that the EST record can serve as a guide, the expression level difference between the two periostin copies in teleosts might be species-specific (zebrafish and stickleback, Additional file 1, Table S1).

It is interesting to speculate that periostin's C-terminal region and its high evolutionary plasticity may explain why periostin, but not TGFBI, is duplicated in teleosts. The absence of a second TGFBI copy in teleosts might reflect the limited freedom of TGFBI to functionally differentiate from the "primary" copy due to the constraints imposed by the EMI and FAS1 domains, leading to loss of the redundant, if not harmful, second copy. By contrast, periostin's C-terminal region could be instrumental for the rapid evolvement of a functional role sufficiently distinct from the periostin paralog in question.

Additional file 6 summarizes further aspects of periostin's C-terminal region in particular and the periostin/TGFBI paralogy in general, placing periostin and TGFBI into a larger framework of chordate evolution.

### Interpretations of the secondary structure prediction results for periostin's C-terminal region

Through secondary structure prediction on periostin's C-terminal region from seven tetrapod and two teleost species, we found consecutive beta strands as the predominant structural feature of this region, specifically, one beta strand per repeat unit. For tetrapods, this means that generally two beta strands are encoded per exon.

The universal splicing variations and the genomic variability observed across and between species could, in principle, point to a functional differentiation of the C-terminal region of periostin. This interpretation is especially attractive in the light of both literature reporting isoform-specific findings and our observation of the eight additional genomic copies of exon 19 in *X. tropicalis*, the expression of which might be tied to developmental stage, specifically (pre)metamorphic animals, according to EST data.

The extracellular matrix is rich in potential interaction and binding partners for periostin, a few of which have been elucidated in some detail. Most prominent among these are a number of integrins (e.g. [29-32]). Heparin binding has been shown, possibly mediated by a Cardin-Weintraub consensus sequence [37,80] encoded by exon 23.

More recently, periostin binding to collagen I was reported in the context of a phenotypic characterization of periostin-null mice [35], but the binding mechanism was not described. Similarly, periostin binding with fibronectin was observed, but not explained on a molecular or structural basis [38].

Since collagen and fibronectin binding have not been described for TGFBI, the periostin paralog lacking an extended and variable C-terminal region, we hypothesize that periostin might interact with these proteins in this region. Collagen I - fibronectin binding has recently been elucidated in complexes of two or four sequential fibronectin FN1 or FN2 domains, components of a fibronectin region called "gelatin-binding domain", with peptides from the collagen I α_1 _or α_2 _chain. With the two fibronectin domains in question being beta-sandwich structures, the collagen peptides assume, in the complex, a beta strand conformation with anti-parallel orientation to one of the FN domain beta strands (beta-zipper) [81].

This mode of binding is similar to earlier descriptions of the way bacterial cell wall proteins from *Staphylococcus aureus *and *Streptococcus pyogenes *attach to human fibronectin [82,83]: Here, bacterial repeats were found to form a tandem beta-zipper with two fibronectin FN1 domains. This finding and the repetitive arrangement of both the bacterial units and the FN1 domains then led the authors to propose a model of an extended beta-zipper, involving a larger number of FN1 domains and bacterial repeats. Therefore, since the periostin's C-terminal region has repeats reminiscent of these bacterial repeats, we hypothesize that they may assume a structure of multiple consecutive beta strands when binding to sequential beta strand elements of fibronectin domains (and possibly other ECM proteins) by way of an extended beta-zipper.

In such a model, the strength of this interaction would be influenced by the number of beta strands available for binding with - in the case of fibronectin - maximally 11 available sequential FN1/FN2 domains in the N-terminal region. The number of available beta strands from periostin's side is a function of exon count, which is in turn determined - and, in this model, calibrated or tuned - by alternative splicing as well as lineage-specific differences.

## Conclusions

We believe that this study marks an important contribution to periostin research. Despite the considerable increase in published literature since periostin's discovery in an osteoblast cell line in 1993, especially over the past few years, some aspects have remained poorly understood. This paper provides evolutionary context for periostin's enigmatic C-terminal region and argues that it is likely key to a detailed understanding and disambiguation of periostin function, which is subject to some controversy in present literature. We put forward a number of findings and interpretations that will hopefully be taken on in further studies and experimentally in the future. We believe that our secondary structure prediction, the finding of additional genomic exon 19 copies in *X. tropicalis*, and the duplication of periostin in teleosts are particularly rewarding experimental targets.

Finally, we note that this work has benefited greatly from the growing collection of whole-genome sequences available, without which it would have been impossible in its present form. At the same time, it strongly supports the argument not only for continued new genome sequencing, especially of strategically selected representatives from key phyla, but importantly also for a sustained effort to finish, at least to a reasonable degree, existing draft assemblies. While it will not be practical in all cases to arrive at high-quality chromosome-by-chromosome assemblies that are subject to sustained curation efforts, highly fragmented genome sequences render detailed gene-level analyses impossible, or at least severely limit the power of their results. Some aspects of our work remained incomplete, mirroring the unfinished state of key genome assemblies, for example within the teleosts, but especially outside the euteleostome group.

## Authors' contributions

SH conceived of and performed most of the analyses. MAA and SH discussed the research on an ongoing basis, and MAA helped with some analyses. SH wrote the manuscript with input from MAA, and both authors approved its final version.

## Supplementary Material

Additional file 1 **Table S1: Periostin in seven tetrapod and five teleost species**. For periostin from seven tetrapod and five teleost species, this table summarizes data on chromosomal location, available transcript abundance and variation, and the periostin protein sequences reconstructed in the course of this work and listed in Additional file 5. Click here for file

Additional file 2 **Supplementary Figure S1**. Genomic sequence alignment of the periostin locus for 15 species and illustrating the variable conservation of exon21V22. Click here for file

Additional file 3 **Supplementary Tables S2 and S3**. Select alignments of genomic sequences comprising periostin exon 21V22 and exon 17 (Tables S2A, S2B) and overview of *Xenopus tropicalis *transcript sequence evidence covering the periostin C-terminal region (Table S3). Click here for file

Additional file 4 **Supplementary Figures S2 and S3**. Matrix dot plots of periostin C-terminal repeats in teleosts (Figure S2) and human and chicken (Figure S3). Click here for file

Additional file 5 **FASTA-formatted sequence listing**. Protein sequences of periostin and certain periostin homologs relevant for this work. Click here for file

Additional file 6 **Additional evolutionary and phylogenetic considerations**. This document summarizes additional evolutionary and phylogenetic considerations regarding periostin and its paralog, TGFBI, based on analyses of periostin/TGFBI homologs outside the euteleostomes. Contains Supplementary Figures S4 - S7. Click here for file
