# Supplementary material for: Periostin shows increased evolutionary plasticity in its alternatively spliced region
Source: BMC Evol Biol. 2010 Jan 28;10:30. doi: 10.1186/1471-2148-10-30 (PMC2824660; doi:10.1186/1471-2148-10-30)
Supplement: Additional file 1 — Table S1: Periostin in seven tetrapod and five teleost species. For periostin from seven tetrapod and five teleost species, this table summarizes data on chromosomal location, available transcript abundance and variation, and the periostin protein sequences reconstructed in the course of this work and listed in Additional file 5. [file 1471-2148-10-30-S1.PDF]

Table S1: Periostin in seven tetrapod and five teleost species

Species, locus (if > 1); UCSC assembly name/date	Chromosomal region	#Refseqs / mRNAs / ESTs	Observed non-canonical exons, alternative splicing (AS) [1] *Comments*	Periostin sequence based on	Missing exons [2]	Periostin protein sequence identifier [3, 4]
**Human (Hs)** (hg18/Mar2006)	chr13:37,034,001-37,082,000	4 / many / many	▪ Alt. 5'-most exon suggested<br>▪ **AS:** exons 17, 18, 19 (weakly supported), 21	NP_006466.1 [R]		>Hs_PNfl_ _NP_006466.1 [i]
**Mouse (Mm)** (mm9/July2007)	chr3:54,164,001-54,196,000	1 / many / many	▪ **AS:** exons 17, 20 (weakly supported), 21	NP_056599.1 [R], ESTs		>Mm_PNfl
**Opossum (Md)** (monDom4/Jan2006)	chr4:301,065,001-301,112,000	0 / none / none	▪ Exon 21V22<br>▪ Shorter version of exon 18	Genomic sequence [H]		>Md_PNfl
**Platypus (Oa)** (ornAna1/Mar2007)	Ultra336:2,915,001-2,966,000	0 / none / none	▪ Exon 21V22	Genomic sequence [H]	23	>Oa_PNfl-ex23
**Chicken (Gg)** (galGal3/May2006)	chr1:176,287,001-176,325,000	1 / some / some	▪ Exon 21V22<br>▪ **AS:** Exons 17, 18, 21, 21V22	NP_001025712.1 [R], ESTs		>Gg-PNfl
**Lizard (Ac)** (anoCar1/Feb2007)	scaffold_65:3,980,001-4,060,000	0 / none / none	▪ Exon 21V22	Genomic sequence [H]	23	>Ac_PNfl-ex23
**Frog (Xt)** (xenTro2/Aug2005)	scaffold_505:99,001-160,000	1 / some / many	▪ Exon 21V22<br>▪ Cluster of 8 genomic exon 19 copies (exons 19A..H)<br>▪ **AS:** Exons 19A..H, 21, 21V22<br>▪ *Appearance of an alt. 3'-most exon likely due to assembly artifact.*<br>▪ *Exon 21 obscured by gap in assembly, but evident in ESTs*	NP_001106376.1 [R], ESTs		>Xt_PNfl<br>>Xt_PNfl+ex19A..H
**Zebrafish (Dr), loc 1** (danRer5/July2007)	chr15:25,151,001-25,183,000	2 / very few / many	▪ Long repeat exon 18=19<br>▪ Additional two 5'-most exons, observed in 5 ESTs<br>▪ **AS:** exons 20, 21, 22<br>▪ *Apparent AS of exon 17 most likely a BLAT mapping artifact*	NP_981966.1 [R], ESTs		>Dr_PNfl.loc01
**Zebrafish (Dr), loc 2** (danRer5/July2007)	chr10:19,780,001-19,840,000	0 / none / very few	▪ Long repeat exon 18=19 (*only partially covered by transcript evidence!*)<br>▪ 6 exons between 18=19 and 23, 4 of which show ~13aa repeat<br>▪ Alternative 5' end suggested	ESTs, genomic sequence [H]		>Dr_PNfl.loc02
**Stickleback (Ga), loc 1** (gasAcu1/Feb2006)	chrI:13,650,001-13,661,000	0 / none / some	▪ Long repeat exon 18=19<br>▪ **AS:** exon 22	ENSGACP00000 015398 [E], ESTs		>Ga_PNfl.loc01
**Stickleback (Ga), loc 2** (gasAcu1/Feb2006)	chrVII:20,256,001-20,268,000	0 / none / some	▪ Long repeat exon 18=19<br>▪ Only 3 exons following exon 18=19	ENSGACP00000 027192 [E], ESTs		>Ga_PNfl.loc02
**Medaka (Ol), loc 1** (oryLat2/Oct2005)	chr13:7,474,001-7,487,000	0 / none / very few	▪ **AS:** exons 17, 18 (as long/short/absent), 19?<br>▪ No repeat exon (18=19) clearly identified.<br>▪ *Possibly only 20 exons total.*	ENSORLP00000 004465 [E], ESTs, genomic sequence [H]	19	>Ol_PNprtl.loc01
**Medaka (Ol), loc 2** (oryLat2/Oct2005)	scaffold4189:1-6,488	0 / none / very few	*Not assessable. POSTN is partly covered by short scaffold4189, which is flanked by assembly gaps.*	n/a		*Not reconstructed!*
T. nigroviridis **(Tn), loc 1** (tetNig1/ Feb2004)	chr16:4887001-4897000	0 / very few / none	▪ Repeat exon 18=19<br>▪ Only 3 exons following exon 18=19 (?)	CAG09019.1 [G]	1,2,7[p], 8	>Tn_PNprtl.loc01_ _CAG09019.1 [i]
T. nigroviridis **(Tn), loc 2** (tetNig1/Feb2004)	chr7:2,694,001-2,709,000	0 / none / none	▪ Long repeat exon 18=19 (*not covered by transcript evidence!*)	GSTENT00018 874001_prot [E], genomic sequence [B]	17	>Tn_PNprtl.loc02
T. rubripes **(Tr), loc 1** (fr2/Oct2004)	chrUn:57,690,001-57,701,000	0 / none / none		NEWSINFRUP00000 152208 [E]	1[p], 16ff	>Tr_PNprtl.loc01__ N.00000152208 [i]
T. rubripes **(Tr), loc 2** (fr2/Oct2004)	chrUn:234,103,001-234,123,000	0 / none / none	▪ Long repeat exon 18=19 (*not covered by transcript evidence!*)	NEWSINFRUP00000 144260 [E]	17, 20ff	>Tr_PNprtl.loc02__ N.00000144260 [i]

[1] Non-canonical exons and alternative splicing based on transcript data as available in UCSC browser. Non-canonical exons relative to periostin exon structure as established for human and mouse (23 exons). Listing of an exon under 'AS' implies alternative presence or absence unless otherwise noted. [2] Missing exons were not identified despite our efforts, were missing from public sequences (Tn loc 1, Tr), or were not incorporated into reconstructed sequence (Ol), but are thought to be present in reality. [3] All sequences provided in Additional file 5. [4] "fl" identifies full-length sequences (missing exon 23 acceptable), "prtl" identifies partial sequences.
[R] RefSeq database, [H] via homology searches, [E] Ensembl database, [G] GenPept database; [p] partial, [i] Identical to public sequence indicated in identifier
