# Supplementary material for: Periostin shows increased evolutionary plasticity in its alternatively spliced region
Source: BMC Evol Biol. 2010 Jan 28;10:30. doi: 10.1186/1471-2148-10-30 (PMC2824660; doi:10.1186/1471-2148-10-30)
Supplement: Additional file 2 — Supplementary Figure S1. Genomic sequence alignment of the periostin locus for 15 species and illustrating the variable conservation of exon21V22. [file 1471-2148-10-30-S2.PDF]

Periostin shows increased evolutionary plasticity in its alternatively spliced region

Sebastian Hoersch and Miguel A. Andrade-Navarro

Additional file 2

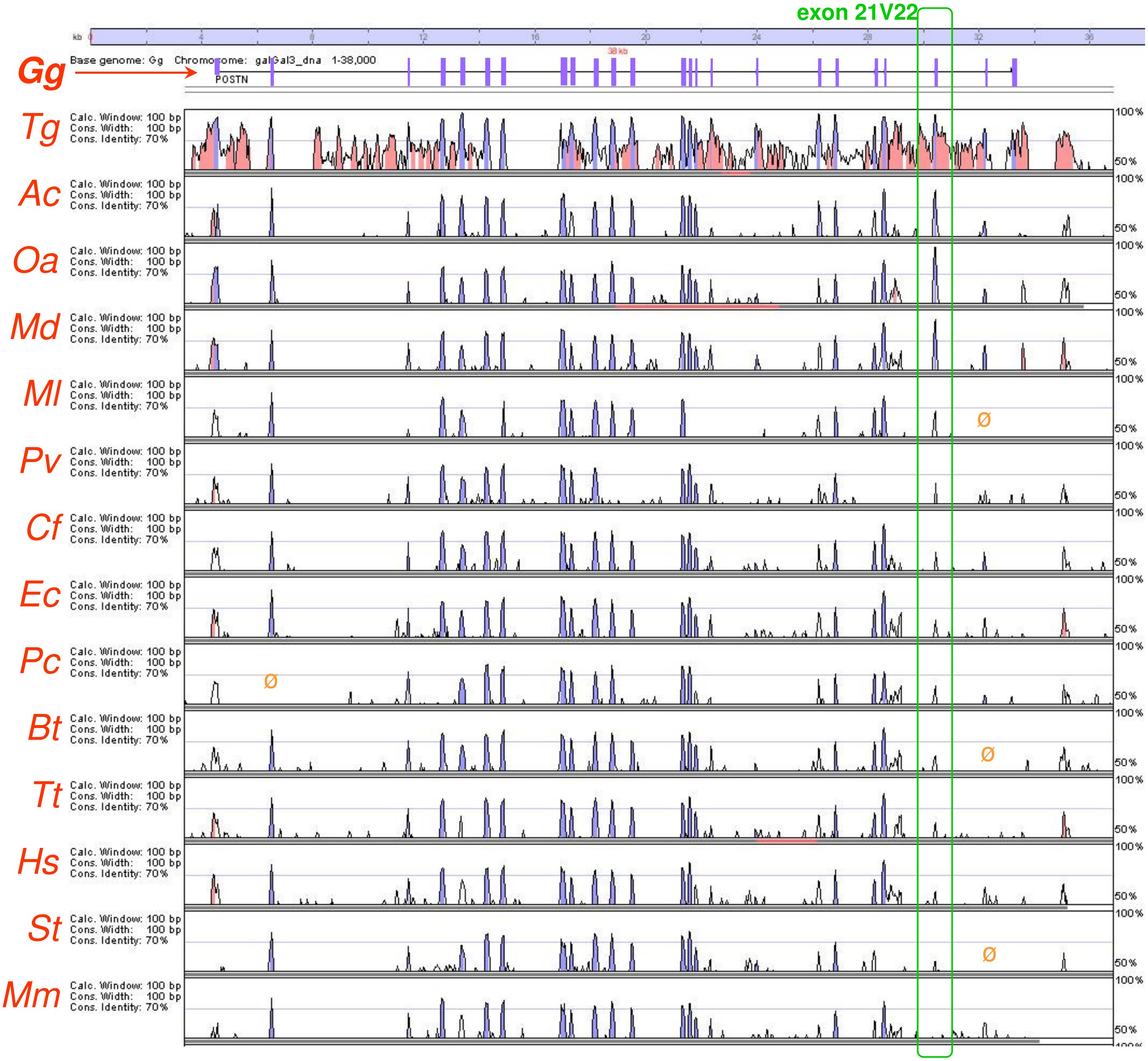

Figure S1: Genomic sequence alignment of the periostin locus of 15 species*) obtained with VISTA. Alignments are relative to chicken periostin (G*g*, top). Conservation curves from 14 other species are displayed below, arranged manually by decreasing conservation of exon 21V22 (green frame). Conservation peaks of exons are colored blue, of non-coding sequence red. While conservation levels stay generally high (above the 70% mark indicated by the faint horizontal line) for most exons or, in some cases, are universally low due to exon shortness (for example, exon 17), the peak for exon 21V22 displays the widest range of conservation level of all exons, from very highly conserved (Tg, Ac, Oa, Md, coming close to the 100% line) to not registering in the VISTA output (Mm, not exceeding the 50% line), with other species on a continuum in-between.

)Gg***: chicken (*Gallus gallus*), ***Tg***: zebra finch (*Taeniopygia guttata*), ***Ac***: anole lizard (*Anolis carolinensis*), ***Oa***: platypus (*Ornithorhynchus anatinus*), ***Md***: opossum (*Monodelphis domestica*), ***Ml***: microbat (*Myotis lucifugus*), ***Pv***: megabat (*Pteropus vampyrus*), ***Cf***: dog (*Canis familiaris*), ***Ec***: horse (*Equus caballus*), ***Pc***: hyrax (*Procavia capensis*), ***Bt***: cow (*Bos taurus*), ***Tt***: dolphin (*Tursiops truncates*), ***Hs***: human (*Homo sapiens*), ***St***: squirrel (*Spermophilus tridecemlineatus*), ***Mm***: mouse (*Mus musculus*).

The **Ø** symbol indicates regions of partially missing genomic sequence.
