# Supplementary material for: Periostin shows increased evolutionary plasticity in its alternatively spliced region
Source: BMC Evol Biol. 2010 Jan 28;10:30. doi: 10.1186/1471-2148-10-30 (PMC2824660; doi:10.1186/1471-2148-10-30)
Supplement: Additional file 3 — Supplementary Tables S2 and S3. Select alignments of genomic sequences comprising periostin exon 21V22 and exon 17 (Tables S2A, S2B) and overview of Xenopus tropicalis transcript sequence evidence covering the periostin C-terminal region (Table S3). [file 1471-2148-10-30-S3.PDF]

Periostin shows increased evolutionary plasticity in its alternatively spliced region
Sebastian Hoersch and Miguel A. Andrade-Navarro

Additional file 3: Tables S2 and S3

Table S2: Select genomic alignments of periostin exon 21V22 and exon 17 produced by VISTA

In the representation below, highly conserved regions were background highlighted by VISTA [63], blue for coding sequence, pink otherwise. Coding sequence for the respective exon was otherwise manually rendered in blue font. Intronic splice sites are highlighted in green, relevant stop codons in red, and the codon corresponding to the central chicken exon21V22 proline in yellow, if conserved, or magenta, if mutated. Translations of exon sequences (bold) are given below the alignment blocks, in red font where frameshifted relative to a functional exon (for exon21V22).

Table S2A: Exon 21V22

```
Top: chicken [>Gg galGal3_dna:30581-30931 (+)]
Bottom: zebra finch [>Tg taeGut1_dna|_RevComp_|:29612-29959 (+)]

000030581  AAGACCACAGCATGATTACTGTGACCTGCATACATCTACACTCCCAAAA-TGACATTCTT
>>>>>>>>>        | ||||||      ||   ||||| ||| |||  ||||||||| ||||||| ||
000029612  ------ATAGCATG------GTCGTCTGCA-ACACCTA--CTCCCAAAAATGACATTGTT

000030640  TCCACTGAAGATTTCT-------------ATGCTATCCCAGATTTAACTCATTTGTTTTT
>>>>>>>>>  ||| |||| |  | ||             ||||||| |||| ||||||||||||||||||
000029657  TCCTCTGAGGGCTCCTTGCATATGGAAAAATGCTATGCCAGCTTTAACTCATTTGTTTTT

000030687  AATGCTTATCATAGCTGGAACTGAGTACACCAAGGTTACTAAAGTAATTGAGGGAGAACC
>>>>>>>>>   ||||||| |||||| ||||||||||||||||||||||||||||||||||||||||| ||
000029717  GATGCTTACCATAGCCGGAACTGAGTACACCAAGGTTACTAAAGTAATTGAGGGAGAGCC

000030747  ACAGATTATCGAGAGAGAAATCAAGAAAGTCCATTTGGAAGGTTAGTTTGTCTATAAATC
>>>>>>>>>  |||||||||||||||||||||||||||||| ||| |||||||||||||| || | ||||
000029777  ACAGATTATCGAGAGAGAAATCAAGAAAGTTCATCTGGAAGGTTAGTTTTTCAA-AAATT

000030807  CAATATCACTAAGGCCTCCTCTGTCTGAAGTAATCATTCTAGGTGCCAATTTATAGTATT
>>>>>>>>>   | |||||||||| | | || ||||  |||| ||||||| |||||    |||||  |||
000029836  AAGTATCACTAAGACTTGCTTTGTCCAAAGTGATCATTCCAGGTGAGGTTTTATCATATC

000030867  TATTCACAAAAGTTACTTAGGTCTGAAGTCAATATTTTAGCAAGAGTTTTATGTTTGATT
>>>>>>>>>  ||||||  ||| ||| ||||||||| || ||  | |||||||||||||||||  ||| ||
000029896  TATTCATTAAAATTA-TTAGGTCTGGAGCCAGCACTTTAGCAAGAGTTTTATAATTGTTT


hoersch@luria:~/bin/Tools/translateDNA.pl -ks -frames rna -minlength 5
>Tg_ex21V22 (len=87)
CCGGAACTGAGTACACCAAGGTTACTAAAGTAATTGAGGGAGAGCCACAGATTATCGAGAGAGAAATCAAGAAAGT
TCATCTGGAAG

>Tg_ex21V22|F3_1|[3-86]
GTEYTKVTKVIEGEPQIIEREIKKVHLE
||||||||||||||||||||||||||||
GTEYTKVTKVIEGEPQIIEREIKKVHLE (Gg)
```

```
Top: chicken [>Gg galGal3_dna:30581-30931 (+)]
Bottom: opossum [>Md monDom4_dna|_RevComp_|:11489-11808 (-)]

000030581  AAG--ACCACAGCA-------------TGATTACTG---TGACCTGCATACATCTACACT
>>>>>>>>>   |   |||| ||||             ||| || ||   |  |   |   ||    | |
000011808  GAATTACCAAAGCACATACACCTTTTTTGAGTAATGTTCTATCTCACTATCACACTCGCC

000030623  CCCAAAATGACATTCTTTCCACT-------GAAGATTTCTATGCTATCCCAGATTTAAC-
>>>>>>>>>   | ||||||       |||||         |||   |||| | | |    | ||| ||
000011748  TCTAAAATGCTCCCAATTCCATCATCCAAGGAAATGTTCTCTCCAAAATTATATTGAATT

000030675  ------------TCATTTGTTTTTAATGCTTATCATAGCTGGAACTGAGTACACCAAGGT
>>>>>>>>>              | | |||||| ||||||||||| |||||||||||||||||||||||||
000011688  GTACAACTGTGATTACTTGTTTCTAATGCTTATCTTAGCTGGAACTGAGTACACCAAGGT

000030723  TACTAAAGTAATTGAGGGAGAACCACAGATTATCGAGAGAGAAATCAAGAAAGTCCATTT
>>>>>>>>>   ||||||||||||||| | ||||||||||||||||| |  ||||||||||||||||
000011628  CACTAAAGTAATTGAGAGTGAACCACAGATTATCGAAACGGAAATCAAGAAAGTCCTCCA

000030783  GGAAGGTTAGTTTGTCTATAAATCCAATATCACTAAGGCCTCCTCTGTCTGAAGTAATCA
>>>>>>>>>  || ||||||||||||||||   || || | | ||||||   |
000011568  GGCAGGTTAGTTTGTCTATTCCTCTAACAGCCCTAAGGTTCC------------------

000030843  TTCTAGGTGCCAATTTATAGTATTTATTCACAAAAGTTACTTAGGTCTGAAGTCAATATT
>>>>>>>>>                                       ||  |     ||| ||||
000011526  -------------------------------------TATCTTCACTTGATGTCA-----


hoersch@luria:~/bin/Tools/translateDNA.pl -ks -frames rna -minlength 5
>Md_ex21V22 (len=87)
CTGGAACTGAGTACACCAAGGTCACTAAAGTAATTGAGAGTGAACCACAGATTATCGAAACGGAAATCAAGAAAGT
CCTCCAGGCAG

>Md_ex21V22|F3_1|[3-86]
GTEYTKVTKVIESEPQIIETEIKKVLQA
|||||||||||| |||||| |||||
GTEYTKVTKVIEGEPQIIEREIKKVHLE (Gg)
```

Top: chicken [>Gg galGal3_dna:30581-30931 (+)]
Bottom: *M. lucifugus* [>little.bat GeneScaffold_2401|_RevComp_|:245049-245249 (+)]

```
000030581  AAGACCACAGCATGATTACTGTGACCTGCATACATCTACACTCCCAAAATGACATTCTTT
>>>>>>>>>    |     |  || || | | |                   || | |||  |||||
000245049  CGGGTACTAATATTATGATTTT--------------------TCACTAAAATACATT----

000030641  CCACTGAAGATTTCT------------ATGCTATCCCAGATTTAACTCATTTGTTTTTAA
>>>>>>>>>         ||||| ||            ||| | || || | |  |   |   |||||| |
000245086  -------AGATTCCTTAACTAAAAAAAATGTTCTCACAAAATGCATAAA---GTTTTTCA

000030689  TGCTTATCATAGCTGGAACTGAGTACACCAAGGTTACTAAAGTAATTGAGGGAGAACCAC
>>>>>>>>>  | |||||| |||    |||||  |  |||||   ||||||||||||| ||| ||||   |
000245136  TTCTTATCTTAGGCCAAACTGCATTTACCAAAAGTACTAAAGTAATTAAGGAAGAATATC

000030749  AGATTATCGAGAGAGAAATCAAGAAAGTCCATTTGGAAGGTTAGTTTGTCTATAAATCCA
>>>>>>>>>  | | ||| |||| || || ||| || |||    |||||||||
000245196  AAACTATGGAGAAAGGAACCAAAAAGGTCA---TGGAAGGTT------------------

000030809  ATATCACTAAGGCCTCCTCTGTCTGAAGTAATCATTCTAGGTGCCAATTTATAGTATTTA
>>>>>>>>>
000000000  ------------------------------------------------------------

000030869  TTCACAAAAGTTACTTAGGTCTGAAGTCAATATTTTAGCAAGAGTTTTATGTTTGATTTG
>>>>>>>>>  || |||||  | |||
000245235  TTGACAAATCTGACT---------------------------------------------
```

```
hoersch@luria:~/bin/Tools/translateDNA.pl -ks -frames rna
>little.bat_ex21V22 (len=84)
GCCAAACTGCATTTACCAAAAGTACTAAAGTAATTAAGGAAGAATATCAAACTATGGAGAAAGGAACCAAAAAGGT
CATGGAAG

>little.bat_ex21V22|F3_1|[3-83]
QTAFTKSTKVIKEEYQTMEKGTKKV-ME
 |  || ||||  | |  |   |||  |
GTEYTKVTKVIEGEPQIIEREIKKVHLE (Gg)
```

Top: chicken [>Gg galGal3_dna:30581-30931 (+)]
Botton: dog [>Cf canFam2_dna:38906-39134 (+)]

```
000030581  AAGACCACAGCATGATTACTGTGACCTGCATACATCTACACTCCCAAAATGACATTCTTT
>>>>>>>>>                                 |||| ||
000038906  -------------------------------ACATTTA----------------------

000030641  CCACTGAAGATTTCTATGCTATCCCAGATTTAACTCATTTGTTTTTAATGCTTATCATAG
>>>>>>>>>                                                        || |||
000038913  ------------------------------------------------------TCTTAG

000030701  CTGGAACTGAGTACACCAAGGTTACTAAAGTAATTGAGGGAGAACCACAGATTATCGAGA
>>>>>>>>>   |  | ||   |  ||  |   |||||||||| || ||||||||   ||   ||| ||||
000038919  GTAAAGCTATATTTACTGAACCTACTAAAGTA-TTAAGGGAGAATATCAACCTATAGAGA

000030761  GAGAAATCAAGAA--AGTCCATTTGGAAGGTTAGTTTGTCTATAAATC---CAATATCAC
>>>>>>>>>   || ||  || ||  ||||  ||| || ||||      |  | |||||   ||||||  |
000038978  AAGGAACAAAAAAGAAGTCATTTTAGATGGTT------TTGACAAATCTGCCAATATAGC

000030816  TAAGGCCTCCTCTGTCTGAAGTAATCATTCTAGGTGCCAATTTATAGTATTTATTCACAA
>>>>>>>>>          ||| |||  | | ||         ||   ||  || |||   |   |
000039032  ATGTAAAGCCTGTGTAGGGAATA---------GGGATCAGCTTGTAGGTCTGGCTTT---

000030876  AAGTTACTTAGGTCTGAAGTCAA--TATTTTAGCAAG---AGTTTTATGTTTGATTTGGA
>>>>>>>>>     | | || ||    || ||||  || || || |||   | | || |  ||| |||
000039080  ---TGATTTGGGCTCTAATTCAAACTACTTAAGTAAGTTAATTCTTCTACTTGGTTTC--
```

```
hoersch@luria:~/bin/Tools/translateDNA.pl -ks -frames rna
>Cf_ex21V22 (len=88)
GTAAAGCTATATTTACTGAACCTACTAAAGTATTAAGGGAGAATATCAACCTATAGAGAAAGGAACAAAAAAGAAG
TCATTTTAGATG
>Cf_ex21V22|F3_1|[3-56]
KAIFTEPTKVLRENINL*
```

Exon 21V22 *continued*

```
Top: chicken [>Gg galGal3_dna:30581-30931 (+)]
Bottom: human [>Hs hg18_dna:43690-44217 (+)]

000030644  CT-------------GAAGATTTCTATGCTATCCCAG-----------------------
>>>>>>>>>  ||             | | ||||||     ||   ||
000043929  CTTTTGGTAATTTGAGTATATTTCTCAATCATTTAAGTCATGACTATTAACATGGCTAAC

000000000  ------------------------------------------------------------
>>>>>>>>>
000043989  TGGTACTTTGAGTACTGATGTTGTGATTTGTCACTGATACACTTCCAAGACCTTAACTGA

000030668  -----------ATTTAACTCATTTGTTTTTAATGCTTATCATAGCTGGAACTGAGTACAC
>>>>>>>>>             ||| ||  |||    ||||   ||||| | |||    || |  | |  |
000044049  AAAAATTTCTCATTAAATGCATAAAATTTTTGGGCTTACCTTAG----AATTATGCATTC

000030717  CAAGGTTACTAAAGTAATTGAGGGAGAACCACAGATTATCGAGAGAGAAATCA----AGA
>>>>>>>>>    |    ||| ||| |||| | ||||||  ||||| |||   || || ||  |    | |
000044105  TGAAACGACTCAAGCAATTAAAGGAGAATAACAGACTATGTCGAAAGGAACAACAACAAA

000030773  AAGTCCATTTGGAAGGTTAGTTTGTCTATAAATCCAATATCACTAAGGCCTCCTCTGTCT
>>>>>>>>>  |||||  ||| |||||||
000044165  AAGTCATTTTTGAAGGTT------------------------------------------

000030833  GAAGTAATCATTCTAGGTGCCAATTTATAGTATTTATTCACAAAAGTTACTTAGGTCTGA
>>>>>>>>>                                      ||| ||||  |
000044183  ------------------------------------TTCGCAAATCT-------------

000030893  AGTCAATATTTTAGCAAGAGTTTTATGTTTGATTTGGAA
>>>>>>>>>   | ||| ||      ||||| || |  ||||
000044194  -GCCAACAT------AAGAGGTTAAGATTTG--------


hoersch@luria:~/bin/Tools/translateDNA.pl -ks -frames rna -minlength 5
>Hs_ex21V22 (len=87)
AATTATGCATTCTGAAACGACTCAAGCAATTAAAGGAGAATAACAGACTATGTCGAAAGGAACAACAACAAAAAGT
CATTTTTGAAG
>Hs_ex21V22|F3_1|[3-86]
LCILKRLKQLKENNRLCRKEQQQKVIFE
```

```
Top: chicken [>Gg galGal3_dna:30581-30931 (+)]
Bottom: mouse [>Mm mm9_dna:27269-27702 (+)]

000030581  AAGACCACAGCATGATTACTGTGACCT-----GCATACATCTACACTCCCAAAATGACAT
>>>>>>>>>    |   |      |||   | ||| ||      ||  ||| | | ||| ||   | |||
000027269  CTGGAGA------GATGGATCTGAGCTTAAGAACACTCAT-TGCTCTCTCAGGGTAACAG

000030636  TCTTTCCACTGAAGATTTCTATGCTATCCCAGATTTAACTCATTTGTTTTTAATGCTTAT
>>>>>>>>>   || |          |||||  |||  | ||||     ||    ||  |||||  |   |
000027322  GCTCT---------GTTTCTCAGCTTCCACAGAGCAGCCTGGACTGCCTTTAACTCCAGT

000030696  CATAG-----CTGGAAC-----TGAGTACACCAAGGTTACTA-----AAGTAATTGAGGG
>>>>>>>>>     ||     |||| |      |  |  | |||  | |||||     || | ||  |
000027373  TCCAGGGGCTCTGGCATCCTTTTCTGGTCTCCACAGGTACTACATACAAATGATGCATAT

000030741  AGAACCACA---------------------------GATTATCGAGAGAAATCAAGAAAG
>>>>>>>>>  | |  ||||                          || |      |||   |  | ||
000027433  AAACTCACACCCATGCACACACATACATACACATAATAAATAGAGGAGGCCTTTAAAAGA

000030776  TCCATTTGGAAGGTTAGTTTGT-CTATAAATCCAATATCACTAAGGCCTCCTCTGTCTGA
>>>>>>>>>  |  | |        |||| ||| | ||  |     || ||    |||||   ||   | |
000027493  TGTACTATAGTAACTAGTGTGTGCGATGTAGTTTCTAGCATCTGGGCCTGAGCTAGATAA

000030835  AGTAA-----TCATTCTAG----------------------------------GTGCCAA
>>>>>>>>>    ||       || ||||||                                  | | ||
000027553  GTTACCTTTCTCTTTCTAGCTCTCTAAATGTTTCTTCTCCCACATCCACTGGAGAGTCAC

000030856  TTTATAGTATT--------------------ATTCACAAAAGTTACTTAGGTCTGAAGTC
>>>>>>>>>  ||  ||  | ||                   |  ||   ||||| |  | || ||     |
000027613  TTCCTAACACTTTCATGTTGAGATATCTGAGAGCCAGGAAAGTAAACTCGGGCT-----C


hoersch@HoerschBook:~/bin/Tools/translateDNA.pl -ks -frame rna
>Mm_exon21V22 (len=131 or 124?)
GGGCTCTGGCATCCTTTTCTGGTCTCCACAGGTACTACATACAAATGATGCATAT
AAACTCACACCCATGCACACACATACATACACATAATAAATAGAGGAGGCCTTTAAAAGA
TGTACTATAGTAACTA

>Mm_exon21V22|F3_1|[3-98]
ALASFSGLHRYYIQMMHINSHPCTHIHTHNK*
>Mm_exon21V22|F2_1|[2-91]
GSGILFWSPQVLHTNDAYKLTPMHTHTYT*
>Mm_exon21V22|F1_1|[1-48]
GLWHPFLVSTGTTYK*
```

Table S2B: Exon 17

```
Top: human [>Hs hg18_dna:36101-36400 (+)]
Bottom: mouse [>Mm mm9_dna:20236-20526 (+)]

000036101  ATCCTGTGCCTTAGTATCCTACAGAGACAAAAAAGAAAAGGAAAAAAAAAAAAAAACCTTT
>>>>>>>>>  | |   |   |||    |  | | |||||| |||||| | |                 ||||
000020236  AGCTGATAAATTAACGCCTGATAGAGACCAAAAAGGACAA----------------CTTT

000036161  GTTCAGGTT---TTTATGTAAATATTTTTCATGTTCCACTTTCTATACTTTCAGCAACTA
>>>>>>>>>   | |  |||   | ||  ||||  |  |||||| ||||||||||||  ||||||||||||
000020280  ATCCGAGTTGTTTCTAGTTAAACTTCGTTCATGCTCCACTTTCTATGTTTTCAGCAACTA

000036218  AAATTATAACCAAAGTTGTGGAACCAAAAATTAAAGTGATTGAAGGCAGTCTTCAGCCTA
>>>>>>>>>  |||||||||||||||| |||||||||||||||||||| ||| ||||||||||||||||||
000020340  AAATTATAACCAAAGTCGTGGAACCAAAAATTAAAGTCATTCAAGGCAGTCTTCAGCCTA

000036278  TTATCAAAACTGAAGGTAAAAATCAATGTTATACATGGTTCCTT---TTATAGTAAAAAC
>>>>>>>>>  |||||||||| |||||||||   |||   | | |||||||||||   || | | ||||||
000020400  TTATCAAAACGGAAGGTAAAG-CCAACACTCTCCATGGTTCCTTACATTCTGGAAAAAAC

000036335  ATCTGTAAAAAT--ACTGATCTCTATGAATGTATCTACATTCTTTTAAAAACTAACATGT
>>>>>>>>>  || |     |||  ||| |||||| ||||| || ||||||||||||  |||||||||||
000020459  ATATCATGTAATCAACTCATCTCTCTGAATATACCTACATTCTTTTTGAAACTAACATGC

hoersch@luria:~/bin/Tools/translateDNA.pl -ks -frames rna -minlength 5
>Mm_ex17 (len=81)
CAACTAAAATTATAACCAAAGTCGTGGAACCAAAAATTAAAGTCATTCAAGGCAGTCTTCAGCCTATTATCAAAAC
GGAAG

>Mm_ex17|F3_1|[3-80] (len=81)
TKIITKVVEPKIKVIQGSLQPIIKTE
||||||||||||||| ||||||||||
TKIITKVVEPKIKVIEGSLQPIIKTE (Hs)
```

```
Top: human [>Hs hg18_dna:36101-36400 (+)]
Bottom: chicken [>Gg galGal3_dna:23299-23440 (+)]

000036101  ATCCTGTGCCTTAGTATCCTACAGAGACAAAAAAGAAAAGGAAAAAAAAAAAAAAACCTTT
>>>>>>>>>    || |    |||  |||                          |  ||  | ||  |
000023299  GACCAGATGATTACCATC--------------------------AGGAATTATAATTTCC

000036161  GTTCAGGTTTTTATGTAAATATTTTTCATGTTCCACTTTCTATACTTTCAGCAACTAAAA
>>>>>>>>>   || ||  |        ||| |  || ||  |||| |||||||||||||||
000023333  ATTAAGAATG------GAATGTGCTTTATACTCCATTTTCTATACTTTCAG---------

000036221  TTATAACCAAAGTTGTGGAACCAAAAATTAAAGTGATTGAAGGCAGTCTTCAGCCTATTA
>>>>>>>>>                          |||||||  | |||||| |||   | |||||||| |
000023378  ------------------------AAATTAACATAATTGAAAGCAACGTACAGCCTATCA

000036281  TCAAAACTGAAGGTAAAAATCAATGTTATACATGGTTCCTTTTATAGTAAAAACATCTGT
>>>>>>>>>  ||| ||  |||||||||               |||  ||||
000023414  TCAGAAAGGAAGGTAAA---------------TGGGGCCTTC------------------

000036341  AAAAATACTGATCTCTATGAATGTATCTACATTCTTTTAAAAACTAACATGTTAAATGTT
>>>>>>>>>
000000000  ------------------------------------------------------------

hoersch@luria:~/bin/Tools/translateDNA.pl -ks -frames rna -minlength 5
>Gg_ex17 (len=48)
AAATTAACATAATTGAAAGCAACGTACAGCCTATCATCAGAAAGGAAG

>Gg_ex17|F3_1|[3-47]
...........INIIESNVQPIIRKE
           |  ||   ||||  |
TKIITKVVEPKIKVIEGSLQPIIKTE (Hs)
```

Table S3: ***X. tropicalis* transcript sequences covering the periostin C-terminal region.**

For all Genbank transcript sequences found in the UCSC genome browser (xenTro2/Aug2005), the table lists accession numbers, library information, and exon structure with respect to the cluster of exons 19A – 19H and to exons 21 and 21V22. With one exception (BC154911), ESTs from embryonic and metamorphic frogs show presence of at least a subset of exons 19A – H (yellow), while ESTs from adult frogs do not (blue). No such obvious correlation is apparent for exons 21 and 21V22, with exon 21V22 occurring in both developing and adult frogs. Exon 21 is observed in developing frogs only, but is not always present.

mRNA/EST accession	Library Info	Exon status re. the exon 19A-H cluster [1]	Exon status re. exons 21, 21V22 [1]
BC154911	NICHD_XGC_tropTail_m / tail, tga strain, metamorphic	19-20	20-22
DT424643	NIH_XGC_tropSki1 / Skin / Adult	19-20	n/a
CR412272	XGC-tailbud / tailbud (stage 28-30)	19-A-B-C^	n/a
CF345375	NICHD_XGC_Swb1N / whole body / 10 month old male	19-20	20-22
DR891672	NIH_XGC_tropTad5 / whole embryo / Tadpole (st. 36-41)	19-A-B-C-D^	n/a
BX739418	XGC-tadpole / tadpole (stage 35-40) /	19-A-B-D-E-F-20	n/a
CX380890	NIH_XGC_tropTad5 / whole embryo / Tadpole (st. 36-41)	19-A-B-C-D-E-F^	n/a
CX409969	NIH_XGC_tropTad5 / whole embryo / Tadpole (st. 36-41)	19-A-20	20-21-22
DT430191	NIH_XGC_tropSki1 / Skin / Adult	19-20	20-21V22-22
DT425263	NIH_XGC_tropSki1 / Skin / Adult	19-20	20-22
BX706184	XGC-tadpole / tadpole (stage 35-40)	^A-B-D-E-F-G-H-20	20-21-22
CX374357	NIH_XGC_tropTad5 / whole embryo / Tadpole (st. 36-41)	^A-B-C-D-E-F-G-H-20	na
CX320728	NIH_XGC_tropTad5 / whole embryo / Tadpole (st. 36-41)	^B-D-E-F-G-H-20	20-21-22
EL700850	NICHD_XGC_tropLimb_m / Limb / Metamorphic	^D-E-F-G-H-20	20-21-21V22-22
BX739786	XGC-tadpole / tadpole (stage 35-40)	^E-F-G-H-20	20-21-22
BX716782	XGC-tadpole / tadpole (stage 35-40)	^F-G-H-20	20-21-22
BX718380	XGC-tadpole / tadpole (stage 35-40)	^G-H-20	20-21-22
EL712494	NICHD_XGC_tropLimb_m / Limb / Metamorphic	^G-H-20	20-21-22
EL846058	NICHD_XGC_trop_25 / whole / Stage 25	^G-H-20	20-21-22
BX719218	XGC-tadpole / tadpole (stage 35-40)	^G-H-20	20-21-22
EL701127	NICHD_XGC_tropLimb_m / Limb / Metamorphic	^G-20	20-21-22
BX740939	XGC-tadpole / tadpole (stage 35-40)	^G-H-20	20-21-22
CR434701	XGC-tailbud / tailbud (stage 28-30)	^G-H-20	20-21-22
EL710444	NICHD_XGC_tropLimb_m / Limb / Metamorphic	^G-H-20	20-21V22-22
BX709491	XGC-tadpole / tadpole (stage 35-40)	^H-20	20-21-22
EL712816	NICHD_XGC_tropLimb_m / Limb / Metamorphic	^H-20	20-21V22-22
BX721294	XGC-tadpole / tadpole (stage 35-40)	^H-20	20-21-22
CX336698	NIH_XGC_tropTad5 / whole embryo / Tadpole (st. 36-41)	^H-20	20-21-22
CX317169	NIH_XGC_tropTad5 / whole embryo / Tadpole (st. 36-41)	^H-20	20-21-22
CX344015	NIH_XGC_tropTad5 / whole embryo / Tadpole (st. 36-41)	n/a	^21V22-22
CF589667	NICHD_XGC_Swb1N / whole body / 10 month old male	n/a	^21V22-22
CX318515	NIH_XGC_tropTad5 / whole embryo / Tadpole (st. 36-41)	n/a	20-21-22
CX346636	NIH_XGC_tropTad5 / whole embryo / Tadpole (st. 36-41)	n/a	20-21-22
CF378276	NICHD_XGC_Swb1N / whole body / 10 month old male	n/a	20-22
CF377029	NICHD_XGC_Swb1N / whole body / 10 month old male	n/a	20-21V22-22
DT431619	NIH_XGC_tropSki1 / Skin / Adult	n/a	20-21V22-22
CX328322	NIH_XGC_tropTad5 / whole embryo / Tadpole (st. 36-41)	n/a	20-21-21V22-22
CX395892	NIH_XGC_tropTad5 / whole embryo / Tadpole (st. 36-41)	n/a	20-21-22
DT431618	NIH_XGC_tropSki1 / Skin / Adult	n/a	^21V22-22

[1] A '^' symbol identifies exons partially covered.
