# Supplementary material for: Periostin shows increased evolutionary plasticity in its alternatively spliced region
Source: BMC Evol Biol. 2010 Jan 28;10:30. doi: 10.1186/1471-2148-10-30 (PMC2824660; doi:10.1186/1471-2148-10-30)

Periostin shows increased evolutionary plasticity in its alternatively spliced region

Sebastian Hoersch and Miguel A. Andrade-Navarro

Additional file 4: Figures S2 and S3

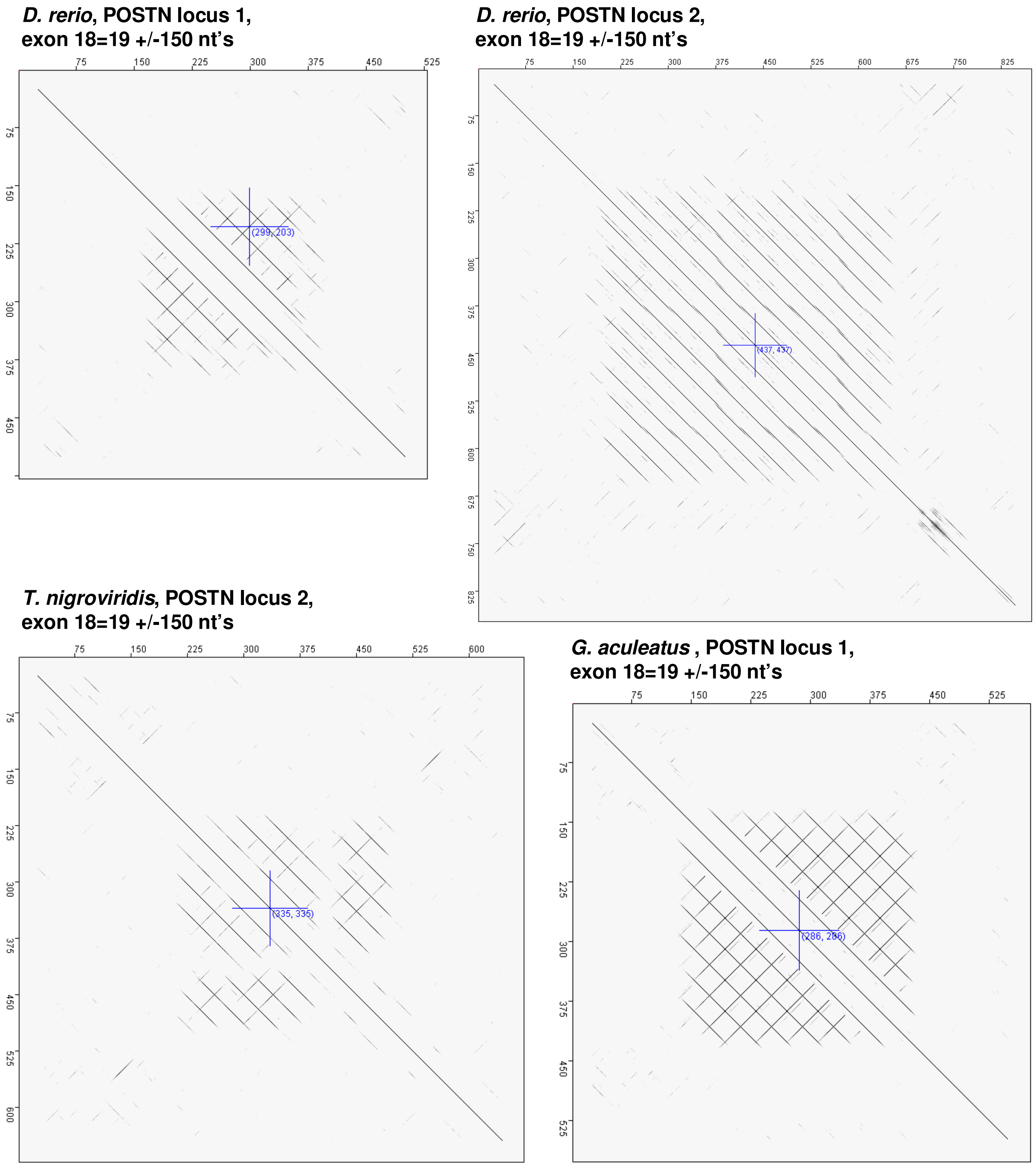

Figure S2: Matrix dot plots of teleost periostin nucleotide sequence comprising exon 18=19 against themselves.

In all four examples, exon 18=19 sequence is flanked by 150 nucleotides of intron sequence on either side. It is clearly visible that the repeat structure does generally not extent beyond the exon boundaries. The varying degree of similarity to the reverse complement repeat sequence is also obvious, being strongest in *G. aculeatus*, locus 1 and basically invisible in *D. rerio*, locus 2.

Figure S3: Matrix dot plots of human (top) and chicken (bottom) periostin nucleotide sequences, exons 15 – 23 against themselves.
Colored boxes delineate exons as indicated in the plots' legends. The repetitive structure and similarities within and among exons 17 and 21 (21V22 for chicken) are clearly visible.

Human periostin, exons 15 – 23

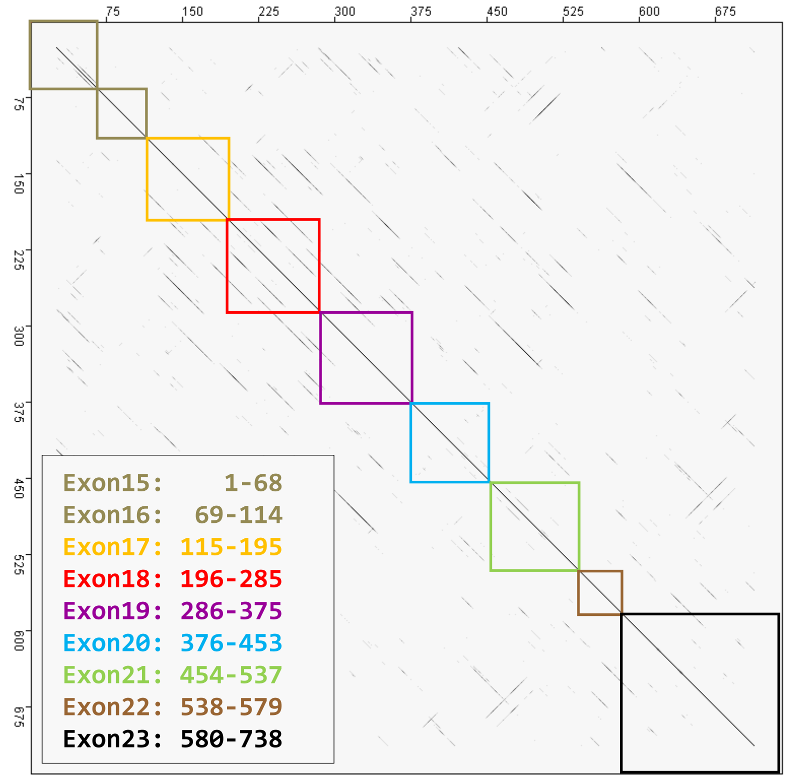

Chicken periostin, exons 15 – 23

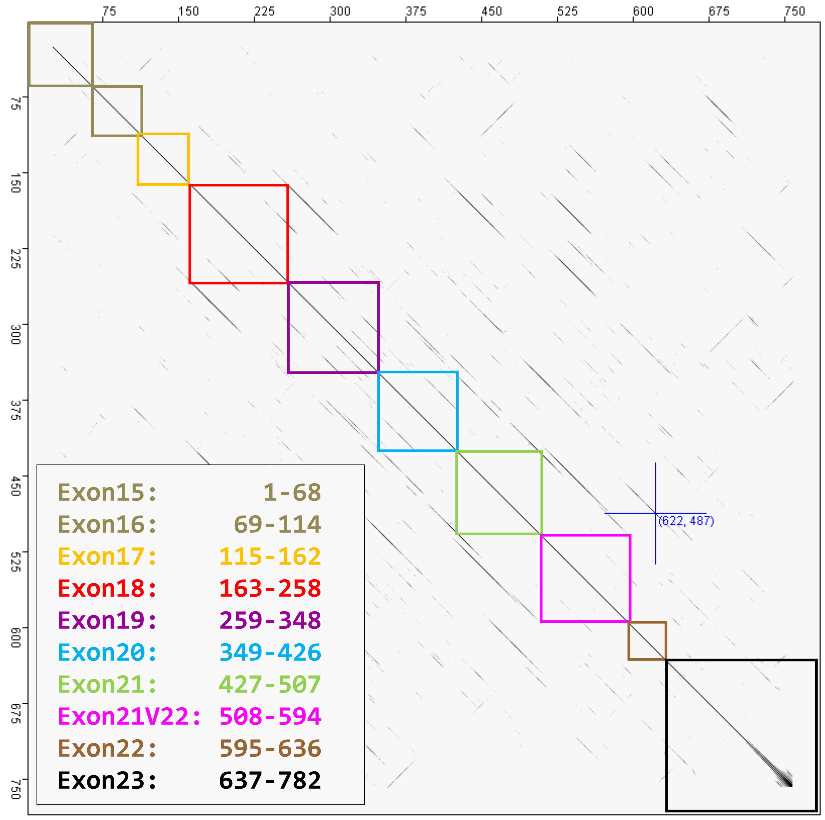

Supplement: Additional file 4 — Supplementary Figures S2 and S3. Matrix dot plots of periostin C-terminal repeats in teleosts (Figure S2) and human and chicken (Figure S3). [file 1471-2148-10-30-S4.PDF]
