# Supplementary material for: Periostin shows increased evolutionary plasticity in its alternatively spliced region
Source: BMC Evol Biol. 2010 Jan 28;10:30. doi: 10.1186/1471-2148-10-30 (PMC2824660; doi:10.1186/1471-2148-10-30)
Supplement: Additional file 5 — FASTA-formatted sequence listing. Protein sequences of periostin and certain periostin homologs relevant for this work. [file 1471-2148-10-30-S5.DOC]

# Periostin shows increased evolutionary plasticity in its alternatively spliced region

Sebastian Hoersch and Miguel A. Andrade-Navarro

# *Additional file 5: protein sequence data*

FASTA-formatted sequence listing
Protein sequence listing of periostin and certain periostin homologs in FASTA format. Sequences that are the result of research conducted in the course of this work are flagged by a red FASTA header, the others list the public accession number in the FASTA header line. With the exception of four translated *Petromyzon marinus* ESTs, sequences are listed as one exon per line (or with indentation if continued from a preceding line) to facilitate ease of use. In the FASTA identifier, the suffix “fl” behind the gene symbol (PN or TGFBI) identifies full-length sequences (missing exon 23 acceptable), the suffix “prtl” identifies partial sequences. Note that amino acids may span exon boundaries. This is not reflected here, and such residues are arbitrarily assigned to one exon.

### Periostin sequences for seven tetrapod and five teleost species (see Additional file 1, Table 1).

**>Hs_PNfl__NP_006466.1 [Homo sapiens]**

MIPFLPMFSLLLLLIVNPINANNHYDKILAHSRIRGRDQG

PNVCALQQILGTKKKYFSTCKNWYKKSICGQKT

TVLYECCPGYMRMEGMKGCPA

VLPIDHVYGTLGIVGATTTQRYSDASKLREEIEGKGSFTYFAPSNEAWDNLDS

DIRRGLESNVNVELLNALHSHMINKRMLTKDLKNGMIIPSMYNNLGLFINHYPNG

VVTVNCARIIHGNQIATNGVVHVIDRVLTQIGTSIQDFIEAEDDLSSFR

AAAITSDILEALGRDGHFTLFAPTNEAFEKLPRGVLERfMGDKVASE

ALMKYHILNTLQCSESIMGGAVFETLEGNTIEIGCDGDSITVNGIKMVNKKDIVTNNGVIHLIDQVLIPD

SAKQVIELAGKQQTTFTDLVAQLGLASALRPDGEYTLLAPVNNAFS

DDTLSMvQRLLKLILQNHILKVKVGLNELYNGQILETIGGKQLRVFVYRT

AVCIENSCMEKGSKQGRNGAIHIFREIIKPAEKSLHEKLKQDKRF

STFLSLLEAADLKELLTQPGDWTLFVPTNDAFKGMTSEEKEILIR

DKNALQNIILYHLTPGVFIGKGFEPGVTNILKTTQGSKIFLKE

VNDTLLVNELKSKESDIMTTNGVIHVVDKLLYPA

DTPVGNDQLLEILNKLIKYIQIK

FVRGSTFKEIPVTVYT

TKIITKVVEPKIKVIEGSLQPIIKTE

GPTLTKVKIEGEPEFRLIKEGETITEVIHG

EPIIKKYTKIIDGVPVEITEKETREERIIT

GPEIKYTRISTGGGETEETLKKLLQE

EVTKVTKFIEGGDGHLFEDEEIKRLLQG

DTPVRKLQANKKVQG

SRRRLREGRSQ

**>Mm_PNfl [Mus musculus]**

MVPLLPLYALLLLFLCDINPANANSYYDKVLAHSRIRGRDQG

PNVCALQQILGTKKKYFSSCKNWYQGAICGKKT

TVLYECCPGYMRMEGMKGCPA

VMPIDHVYGTLGIVGATTTQHYSDVSKLREEIEGKGSYTYFAPSNEAWENLDS

DIRRGLENNVNVELLNALHSHMVNKRMLTKDLKHGMVIPSMYNNLGLFINHYPNG

VVTVNCARVIHGNQIATNGVVHVIDRVLTQIGTSIQDFLEAEDDLSSFR

AAAITSDLLESLGRDGHFTLFAPTNEAFEKLPRGVLERIMGDKVASE

ALMKYHILNTLQCSEAITGGAVFETMEGNTIEIGCEGDSISINGIKMVNKKDIVTKNGVIHLIDEVLIPDS

AKQVIELAGKQQTTFTDLVAQLGLASSLKPDGEYTLLAPVNNAFS

DDTLSMDQRLLKLILQNHILKVKVGLSDLYNGQILETIGGKQLRVFVYRT

AICIENSCMVRGSKQGRNGAIHIFREIIQPAEKSLHDKLRQDKRF

SIFLSLLEAADLKDLLTQPGDWTLFAPTNDAFKGMTSEERELLI

GDKNALQNIILYHLTPGVYIGKGFEPGVTNILKTTQGSKIYLKG

VNETLLVNELKSKESDIMTTNGVIHVVDKLLYPA

DIPVGNDQLLELLNKLIKYIQIK

FVRGSTFKEIPMTVY

TKIITKVVEPKIKVIQGSLQPIIKTER

PAMTKIQIEGDPDFRLIKEGETVTEVIHG

EPVIKKYTKIIDGVPVEITEKQTREERIIT

GPEIKYTRISTGGGETGETLQKFLQK

EVSKVTKFIEGGDGHLFEDEEIKRLLQG

DTPAKKIPANKRVQG

PRRRSREGRSQ

**>Md_PNfl [Monodelphis domesticus]**

MILLLPIFFTLLLSALELADSTAHYDKILLHSRIRGQKQG

PNVCALQQILGTKKKYFSTCRNWYQGAICGKKT

TVLYECCPGYMKMEGMEGCPA

VLPIDHVYGTLGIVGATTTQGYADVSKLREEIEGKGSYTFFAPSNEAWDNLDS

EIRRGLESNVNVELLNALHSHMVNKRMLTKDLKNGMVVPSMYNNLGLFINHYPNG

VVTVNCARIIHGNQIATNGVVHVIDRVLTQIGTSIQDFLEAEDDLSSFR

AAAITSDLLEALGRDGHFTLFAPTNEAFEKLPRGVLERIMGDKVASE

ALLKYHILNTLQCSEAIMGGAVFETLEGHTVEIGCDGESLTVNGVKMVNRKDIVTNNGVIHLIDQVLIPD

TAKQVIELAGKQQTTFTDLMAQLGLASHLKPDGEYTLLAPTNNAFT

DDTLRMDQRLLKLILQNHILKVKVGLNELYNGQHLETLGGKQLRVFVYRT

AICIENSCMLRGSKQGRNGAIHVFQEIIKPAEKSLHETLKQDKRF

SIFLNLLEAADLKDTLIQPGEWTLFVPTNDAFKGMTNEEKEILIR

DKNALQNIILYHLTPGVFIGKDLNLESQTFLRQPKEAKFSLKE

VNNTLLVNEVKSKESDVMTTNGVIHVVDKLLYPA

DLPVGNDQLLEILNKLIKYIQIK

FVRGSTFKEIPVTVYS

TKIITKVVEPKIKVIEGSLQPIVKSE

PFEGNSEFQVIKEGETITKVIHG

EPIIKKYTKIIGGRPVEITEKETTEERIIQ

GPEIKYTRISSGNVGETEETLKKLLQE

EVTKVTKFIEGGDAHLLEDEEIKRLLQG

AGTEYTKVTKVIESEPQIIETEIKKVLQA

DRPVRKVQANKRTQG

TRRRSREGRPQ

**>Oa_PNfl-ex23 [Ornithorhynchus anatinus]**

MKILVLSLFATFLLSVFELADANAHYDKILSHSRIRGREQG

PNVCALQQILGTKKKYFSTCRNWYQGAICGKKT

TVLYECCPGYMRMEGMKGCPA

VAPIDHVYGTLGIVGATTTQGYSDVSKLREEIEGKGSYTYFAPSNEAWDNLDS

DIRKGLESNVNVELLNALHSHMVNKRMLTKDLKNGMIVPSMYNNLGLFINHYPNG

VVTVNCARVIHGNQIATNGVVHVIDRVLTQIGTSIQDFLEAEDDLSSFR

AAAITSDTLEALGRDGHFTLFAPTNEAFEKLPRGVLERIMGDKVASE

ALMKYHILNTLQCSEAIMGGAVFETLEGTTVELGCDGDSLTVNGVKMVNRKDIVTNNGVIHLIDQVLIPD

SAKQVIELAGNQQTTFTDLVAQLGLASSLKPEGEYTLLAPVNNAFT

EDTLRMDERLLKLILQNHILKVKVGLNELYNGQHLETLGGKKLRVFVYRT

AVCIENSCMVRGSKQGRNGAIHVFREIIKPAEKSLHETLKLDKRF

SIFLSLLEAADLKDLLTQPGDWTLFVPTNEAFKGLTNEEKEILIS

DKNALQNIILYHLTPGVFIGKGFEPGVTNVLKTTQGSKIYVKG

VNDTLLINELKTKESDIMTTNGVIHVVDKLLYPA

DTPVGNNRLLEILNKLIKYIQIK

FVHGSNFKEIPLTVYS

TKIITKVVEPKIKVIEGSLQPIIKNE

GPAITKVTVVEGEPEFSLVNEGERITQVIHG

EPIVKKYTKIIGGRPVGITETEVTEERIVK

GPEIKYTRITAAGTAETEEAIQKLLEE

EVTKVTKFIEGGDTHVFEDEEIKRLLQG

AGTEYTKVTKVIEGEPQIVEREIKKVLLE

DQPVKKVQANKRIPG

**>Gg-PNfl [Gallus gallus]**

MKIFLLFTFSTFFLSAFEQAAASAHYDKILTHSRIRARDQG

PNVCALQQVMGTKKKYFSTCRNWYQGSICGKKA

TVLYECCPGYMKMDGMRGCPA

VvPIDHVYGTLGIVGATSTQQYSDvSKLREEIEGRGSFTFFAPSNEAWEQLSS

EIHRNLIDNVNIELYNALHHHMVNKRMLTKDLKNGMTLVSMYNGQKLLINHYPNG

VVTVNCARIIHGNQIATNGVVHVIDRVLTAVGNTIQDFIEVEDDLSSLR

AAAITSDVLDTLGRPGYYTLFAPTNEAFERLPRGILERIMGDKVASE

ALVKFHILNTLQCSEAIMGGAVYETLEGNTLEVGCDGETLTVNGVKMVKRKDIVTSNGVIHLIDRVLIPD

SAKQVIELGGAQQTTFTDLVTQLGLASSLRPEGQYTLLVPQNRAFS

DDTLQMDQRLLKTILQNHIIKVKIGLNELYNGQELETIGGKLLRVFVYRT

AVCVENSCMVRGSKEGRNGFIHVFRQIIKPAEKSLHEMLRNDKRF

SVFLSLVKAADLDDVLSRPGAWTLFVPTNDAFKGLTDDDKAVLIr

DKNALRNILLYHLTQGVFIGSGFEPGVTNILKTIQGGKLYLKT

VNDTLLVNDLKSREPDLMATNGVIHVIDKLLYPA

DLPVGNDQLLTILKKLIKYIQIK

FVRDSTFKEIPLTFYn

KINIIESNVQPIIRKED

PSITQLTKIIEGEPEFKIVREGETITKVIHG

EPIIKTYTKIIDGRPVEVTEKKVTEERIIQ

GPEIKYTRITAGGTDNEENLKRLLEE

EVTKVTKFIEGDAHLLEDEEIKRLLQG

AGTEYTKVTKVIEGEPQIIEREIKKVrLE

EAPVRKVQANKRTQG

GSARRRTRLAYS

**>Ac_PNfl-ex23 [Anolis carolinensis]**

MELLWVAAVSICFLSAFHPAEFATAHYDKILSHSRIRAREQG

PNVCALQQVMGTNKKYFSTCRNWYQGAICGKKA

TVLYECCPGYMKMEGERGCPA

VAPIDHVYGTLGIVGATATQRYSDMSKLREEIEGPGSYTFFAPSNEAWDLLDR

EIHSGLIENVNIELYNALHNHMINKRMLTKDLRNGMTLVSMYNNQNLHINHYPNG

VVTVNCARVIHGNQIATNGVVHVIDRVLTPVGNTIQDFLEVEDDLSSLR

AAAFTSDVMDVLGKPGHYTLFAPTNEAFEKLPRGVLERIMGDKVASE

ALMKFHILNSLQCSEAITGGASFETMEGNTVEVGCDGESLTINGVKMVSRKDIVTSNGVIHLIDQVLIPDS

AKQVIELAGPQQATFKDLVSQVGLAASLRPEEEYTLLAPLNGAFS

DDTLTMDQRILKLILQNHILKVKIGLNDLYNGQFLETLGGKKLRVFVYRT

AVCVENSCMIRGSKEGRNGFIHVFRQLIQPAEKTLHETLRSDKRF

SIFLSLVEAANLEEVLSQPGDWTLFVPTNDAFKGLTEQEKQTLMr

DQNALRNILLYHLTRGVFIGSGFEPGVTNILKTIQGGKLYLKV

VNDSLLVNELKSKESDLMATNGVIHVIDKLLYPA

DMPVGNDQLLAILKKLIKYIQIK

FVRGSTFKEIPMTFYt

TRIITKVVDPKIKVIEGSFEPILGNE

VPSITKITKVIEGEPEFKLVREGETVSKVIHG

EPKIKKYTKIIDGFPVEVTEKEVTEERIIQ

GPELKYTHITTGDGADAEETLKKLLEE

EVTKVTKLIEGDGHLLEDEEIKRFLQG

AGSEYTKVTKVIQGEPKVVETEIKKIHLE

etpvrrvqtsrrtq

**>Xt_PNfl [Xenopus tropicalis]**

MMKGLFLCVFATFLLSAIDHAEGNAYYDKILTHSRIRARQQG

PNVCALQQVLGTKKKYFSTCKNWYQGAICGKKA

TILYECCPGYMKINGESGCPA

VAPIDNVYGTLGIVGATSTQDYSDRSELRKEIEGVGSYTFFAPSNDAWQLLDS

DVRDSLLSNVNIELLNALHYHMINKRMLTKDLKNGLSVTSMFNNQELVINHYTNG

VVTVNCARVIHGNQIATNGVVHVIDRVVTAVGNTIEDFIESEDELTSFR

EagvaaevlaelgkkgqytlfaPtndafeklPrgvlerimadkqavk

ALVNYHILNSVQCSEAIMGGSLLETLEGSSLQIGCDGDSLTVNGNKMVNRKDIVTTNGVIHLIDQVLIPDS

AKQVLELAGSEQTTFTDLMAQMGLAASFRPDAEYTLLAPINNAFS

DETLKMDQRILKLILQNHVLKTKVALNGLYNGQTLETLGGKLLRVFVYRT

AVCIENSCMLRGSKEGRNGAIHIFSEIIKPAEKSFYDLLSLDKRF

SIFLNLIEFAGLKDLLLQPGAWTWFIPTNDAFKGLSNEEMEILKR

DKVALQNILLYHLAPEVFIGGGFEPGVTNILKSLQGNKIMVKAA

VNNTLSVNGVVSKEPDQMTTNGVFHVIDKLLFPA

DVSLGNEQLLSILNKIIKYITIK

FSRGSTFKEIPLTKY

IkFitIgEPRV

TTVTRIIEIKPEMRVVGGETITKVIHG

DPSITRITKVIEGDPEFKLIKEGETRVTKVIQG

GPEITYTRISGLDPDADEETIKKMLEG

ITRVTKFTEGDSQILEDDELKALLQG

GTHISKVTTVHEKEIPETVQISKIVK

EPQVRKVQTGRRTQ

vrrkmrrshqPs

**>Xt_PNfl+ex19A..H [Xenopus tropicalis]**

MMKGLFLCVFATFLLSAIDHAEGNAYYDKILTHSRIRARQQG

PNVCALQQVLGTKKKYFSTCKNWYQGAICGKKA

TILYECCPGYMKINGESGCPA

VAPIDNVYGTLGIVGATSTQDYSDRSELRKEIEGVGSYTFFAPSNDAWQLLDS

DVRDSLLSNVNIELLNALHYHMINKRMLTKDLKNGLSVTSMFNNQELVINHYTNG

VVTVNCARVIHGNQIATNGVVHVIDRVVTAVGNTIEDFIESEDELTSFR

EagvaaevlaelgkkgqytlfaPtndafeklPrgvlerimadkqavk

ALVNYHILNSVQCSEAIMGGSLLETLEGSSLQIGCDGDSLTVNGNKMVNRKDIVTTNGVIHLIDQVLIPDS

AKQVLELAGSEQTTFTDLMAQMGLAASFRPDAEYTLLAPINNAFS

DETLKMDQRILKLILQNHVLKTKVALNGLYNGQTLETLGGKLLRVFVYRT

AVCIENSCMLRGSKEGRNGAIHIFSEIIKPAEKSFYDLLSLDKRF

SIFLNLIEFAGLKDLLLQPGAWTWFIPTNDAFKGLSNEEMEILKR

DKVALQNILLYHLAPEVFIGGGFEPGVTNILKSLQGNKIMVKAA

VNNTLSVNGVVSKEPDQMTTNGVFHVIDKLLFPA

DVSLGNEQLLSILNKIIKYITIK

FSRGSTFKEIPLTKY

IkFitIgEPRV

TTVTRIIEIKPEMRVVGGETITKVIHG

DPSITRITKVIEGDPEFKLIKEGETRVTKVIQG

EPTITKITRVIEGEPEFKLIKEGETRVTKVIQG

EPTITKITRVIEGEPEFKLIKEGETRVTKVIQG

EPTLTRITKVIGGESDLHLVKEGETRVTKVIQG

EPTLTRITKVIGGEPDFHLVKEGETRVTKVIQG

EPITRITKMIEGDQDFHLVREGETRVTKVIQG

EPSITKITRVIEGEPDFKLIREGETRVTKVIQG

EPTITKITRVIEGEPEFKLIKEGETRVTKVIQG

EPIITKLTRVVEGEPDFRLIKEGETRVTKVIQG

GPEITYTRISGLDPDADEETIKKMLEG

ITRVTKFTEGDSQILEDDELKALLQG

GTHISKVTTVHEKEIPETVQISKIVK

EPQVRKVQTGRRTQ

vrrkmrrshqPs

**>Dr_PNfl.loc01 [Danio rerio]**

MKLLFAATFALFVLSAFDQADSSAYDKIVAHSRIRAKKQG

PNMCALQQVVGTKKKYFSTCRNWYQGAICGKTA

TVLYECCPGYMELAGQRGCPA

VAPIDNVFGTLGLVKAKTTQDYSDISKLRQEIEGAGSYTFFAPSNDAWDLLEA

EVRNALVSNVNIELYNALHYHMVNKRLLTKDLKNGMTATSMYNDLSLHINHYSNG

VVTVNCARIIHGNQVATNGVVHVIDRVITAVGSTIQDMIEVEDDLSTLS

TVATDSGLIDKLGEPGHFTLFAPTNAAFDKLGREVLDRLMKDKKSLQ

ALLNYHLLNSVQCSEAIMAGTSHETLEGSNIEIGCDGDSLTVNGIKMVLKKDIVATNGVIHLIDEVLMPDS

AKQVMELVGQSQATFSDMLTELGLSAAMRPQAEYTLLAPVNAAFN

DEVMSMDQSFLKIILENHILKNKIVLSQLYNGQRLETLSGKFLRVFVYRT

ALCIENACLIRGSKEGSNGALHLMKTLISPAESSMFQILVKNGAFK

IFLSLMEAAGLTDLLKQEGDFTLFAPTDEAFAGLSERDLTLLKS

NGNALKAILLYHFSNGVFIGGGLETGVTNLLKTLQGSNLRVLY

ANASMLVNTVKVPEPDIMATNGVVHFVRTLLYPE

DIPVGSQDLLSLLRRIIRYIQFK

FVPGYRYQEIPLTFM

rrvit

VPGDVTKVTRVIQGEPTITKVTRVIEGAPSVTKVTRVIEGQPSITKVTRVIEGQPSLTKVTRVIEGDPSMTRVIE

GPEYSVTSSTDTELY

DFGEDSEGITTFI

QEGTRRVPPRRVQG

gvrrrt

**>Dr_PNfl.loc02 [Danio rerio]**

MILLFTATFTILVLSSLDQVDSSAYDKIVSHSRIRAKNEG

PNVCALQQVMGTKKKYFSTCRNWYKKSICGKKA

IVLYECCPGYMKLEGKRGCPA

VAPIDSVYGTLDLVKAKTTQQYADQSKLREEIAGEGSYTIFAPSDDAWEELD

ASKAAVISLGNTELYNALHYHMVSKRFLTKDLKNDMTLESMFNKQGLHINHYSNG

VVTVNCARIIHGNQVATNGVVHVIDRVISVVSQTIKDVIETNDDLSSLS

GVVVSADLQDQLGEPGHYTLFAPTNEAFDKINADALERLMSDQTVIQ

ALLKYHLLNSVQCSEAIMAGSIYGTLEGSNIEIGCDGESLTVNGIKMVLKKDIVTSNGVIHLIDQVLMPDS

AKQVMELVGKSQSVFSDMVSELGLSAALQPETEYTVLAPLNGAFS

DEVMSMDQRLLKIILENHIVKLKVSLRDLYNGQLLETLGGKMLRVFIYRT

AVCIENACMVRGSREGSNGALHLMSSLIQMPETTIYELLLKDGRFK

IFLSLMESAGLTDLLKQDGSYTLFAPIDAAFGSLTKDDLALLKS

DINVLRTILLYHFSNGIFINGGLEGGVTNLLKTIQGNNLQVLS

VNSSIHVNSVDVPDFDLMASNGVVHVVKTLLYPQ

DLPVGREDILILLKRLIKYMQLK

FVSGYTYRDIPLTFI

KRTITTHVIER

GPGTTVERTVIGEPIKVTRVIEGQPGVTKVTRVIEGDPSVTKVTRVVEGQPGITKVTRVIEGDPSFTKVTRVVGDQSLTKVTRVVEGDPRFTKVTRVVEDDPSLTKVTRVVEGEPSFTKVTRVVEGDPSFTKVTRVVGDPSLSKVTRVVEGKPSVTKVTRLIECMSKLPMINFIITNPCYLITFLSF

ISNVQRFQNGEVHILQEEDIKQITDAIAQG

GGPGITTITRVLKPEPRVVES

EPGITTFTRVIKKKPQIVD

GAPLETTFTRVIKPEPQIIE

GPDFSKIVSLKDNPELFESETERITRIIK

DGRSRKRAAIRQLQG

srrrarlvrhptkptq

**>Ga_PNfl.loc01 [Gasterosteus aculeatus]**

MKLLFLAAFALFVLSTFDHVESSAYDKIVAHSRIRARKE

GPNVCALQQVMGTKKKYFSTCRNWYQGAICGKKA

TVLYECCPGYMKLEGMRGCPA

VAPIDHVYGTLGLVKATSTQKYSDISKLRPEIEGSGSFTIFAPSNDAWELLDE

EVRSALVSNVNIELYNALHYHMANKRLFTKDLRNGMTVTSMYNDLGLQINHYSNG

VVTVNCARIIHANQVATNGVVHVLDRVISSVGNTIQEVVDIEDELTTLS

DVAQNSGIMEKLGQPGHYTLFAPTNEAFESLGSEVLERLQGDKEVLK

ALLNYHLLDSVQCSEAIMTGTSYETMEGNNIEIGCDGESLTVNGIKMVLKKDIVTTNGVIHLIDRVLMPDS

AKQVMELMGSSQSTFGDLVSELGFSAAMRGGAEYTLLAPLNVAFS

DEVMSMDQSLLRIILENHILKRKIVLGELFNGQRLETIAGKYLRVFIYRT

AVCIENSCLVRGSKEGSNGALHLMKILLNPAEKSMFEILREKGGFK

IFLSLMEAAGLTDLLKQEGEFTLFAPSDKAFAGLSQTDLTLLKS

DMNALRTILLYHFNNGIVIGGGLETGVTNLLKSLQGSNLRVMS

ANNSMLVNSVQVPASDIMATNGVIHFVNQVLYPG

DIPVGSQDLLTLLKRLVTYMQIK

YISGFRYKEIPLTFL

RRIITRVVQE

GEPTITKVTRVIEGQPSITKVTRVIEGQPSITKVTRVIEGEPSITKVTRVIEGEPSITKVTRVVEGQPSITKVTRVIEGQPSITKVTRVIE

GPEFSVSSGTTNIALE

AANISDMDDDSKRLVQIIQ

EGSSRRTASRRVVD

SNRRRGRD

**>Ga_PNfl.loc02 [Gasterosteus aculeatus]**

MHQLLVVTSVLVALCSLGSVDTSAYDKIVTHSRIRARKE

GPNVCALQQVQGSKKKYFSTCRNWYKGSICGKKT

LVLYECCPGYIKLEGMRGCPA

VAPIDHVYGTLGLIKATTTQQYSDTAKLREEIEGQGSFTMFAPSNDAWDLVDS

TVRSALVSNVNIELYNALHFHMVNRRILTKDMKNDMTFTSMYNNLGLYVNHYSNG

IVTVNCARIVLGNQVATNGVVHVIDRVISSVSNNIKEVLDVTDDLSAFT

NVVMASGMMDKLDQPGHYTLFAPTNEAFDKLSPGYLERIMGDKAVIA

ALVNYHLLSSVQCSEAIMSGTAYETAEGSTIEIGCDGDSLTVNGITMVLKKDVVTTNGVIHFIDQVLIPDS

AKEGMELMGDSQSTFSNMLYEMGFSAALGPKTEYTLLAPLDSAFT

HEVTDKNVLKFLLQNHILKMKVTLSELYNGQTLETVAGKLLRVFIYRT

AVCIENACMVRGSKEGSNSALHVMRTLIKPPEKTIYELLIADRRFK

IFLSLIETAGLTNLLKQEGSYTIFAPTDDAFDNLSKEELALLKS

DLNALRTILLYHFSNGIFINGGLERGVTNLLKTFQGKNLQVI

QVNNSIHVNSVDVPNSDLMATNGVVHVVKNVLYPA

DLPVGRQDLLVLLKKLIKYIHIT

YTSGFTYEEIPLTFIR

TTVTTTHIKT

EPTFTKVTKVFQTDPVITEVVVKGEPAITKVTRVIEGDPSFTKVTRVIAGEPSVTKVTRVIEGGDRDRDGKLIA

GADFSKITTIHGNPNRVDEESERITKLIR

DGGGFAAARKAA

VGMRKRRKQLVRRHPKPRE

**>Ol_PNprtl.loc01 [Oryzias latipes]**

MKLLLAAALFVLYTFDSADSSAYDKILTHSRIRAKKEG

PNVCALQQVMGTKKKYFSTCRNWYHGAICGKKA

TVLYECCPGYIKLDGMRGCPAV

APIDHVYGSLGIVKASSTQKYADISKVRPEIEGSGSFTFFAPSNEAWELLDE

DVRNALVSNVNIELYNALHYHMANKRLLTKDLKNGMTITSMYNDLDLHINHYSNG

VVTVNCARIISGNQIATNGVVHVIDRVISAIGNSMQDVIEVEDDLTTLS

DLAQTSGLLEKLGQPGHYTLFAPTNDAFDKLGSDVLERLQSDKEVLK

ALLSFHLLDSVQCSEAIMVGSSYETLEGNSIEIGCDGDSLTVNGVKMVLKKDIVTKNGVIHLIDQVLLPDS

AKQVMELLGSSQSTFGDMISELGISTSMASDAEYTFLAPLNDVFT

DEVMSIDQDDLRVILENHVFKNKIMLGQLYNGQLLETIAGKTLRVFIYRT

AVCIENSCLIRGTKEGANGALHLMKTLLKPAAKTMFEILKQNGNF

KIFLSLMEAAGLTDVLRQEGSFTLFAPSDKAFASLATRDLELLKS

NKNALKTILLYHLTNAVFVSGGLEVGVTNLLKSLQGSSLKLIF

ANSTTQVNSVKVPEADIMATNGVVHFVNQVLYPE

DMPVGSQDLLMVLRKLVTYIQVK

YIAGFKYQEIPLTFM

KTIVTHVVK

EGEPSTIMTTRVVKSGSKPRLFKEEAFSTGRVT

GDVGEEEPWF

**>Tn_PNprtl.loc01__CAG09019.1 [Tetraodon nigroviridis]**

TVLYECCPGYMKLDGMHGCPA

VAPIDHVYGTLGVVKATSTQRYSDISKLRAEIEGPGSYTFFAPSNEAWDALD

QTTTNALVNNVNVELYNALHFHMSNRRLLTKDLKNGMTVTSMYNDLGLYINHYPNG

VVTVNCARIIQGNQVATNGAVHVIDRVISAVGTTIQDFIEVDEDLSTFN

EVAQSAGPLDKLGQPGHYTL

LFLFCFCIFPPILAKQVMELVGDSQSTFRDMVAQLGLSTAMKPKADYTLLAPLNVAF

SDDIMSKDQKLLRIILENHIMKNKVVLGQLYNGQHLETIGGKRLRVFIYRT

AVCIENSCLIRGSKEGSNGALHITKTLLRPAEKSMYEILMENGRFK

IFLSLMEAADLTNLLKQEGDFTLFAPSDKAFAGLTESDLTLLKS

DKNALRTILLYHFNKGIFIGGGLENGVTNIIKSLQGNNIKVIF

ANRTMQVNSLQIPESDIMSTNGVIHFVNHIMYPG

DIPVGNQNLLKLLKKLITNIQIK

YTSGYRYQEIPLTFL

Krvvqe

GANITTLTRVIQSKPSFTKMTIEGIDDFQPSITKIAKVVQ

GPQYSSSAGISTINLE

Drssrrtttrt

sw

**>Tn_PNprtl.loc02 [Tetraodon nigroviridis]**

YDKIVSHSRIRARKEG

PNVCALQQVMGTKKKYFSTCRNWYKGTICGKKA

QVLYECCPGYMKLDSMKGCPA

VAPIDNVYATLGTLHATSTQRYSSMAGMREKLEGMGSFTIFAPSNDAWTELDS

ATRNSLEGNVNTELRNALHYHMANRRLLTKDLKNDITVPSMYNKLGLYINHYSNG

VVTVNCARIIHANQVATNGVVHVIDRVISGVGKTIKDVLDEKDELSSFS

AAALASGIMDKLGRPGHYTLFAPTNEAFENLSPGHLERMMDEKDVIA

ALVNYHLLNSAQCSEAIMAGSVFETAEGSTIEIGCDGDSLTVNGIKMVLKKDIVTTNGIIHLIRRVLIPDA

AKEGLELLGDSQMKFSNMVSEVGLAAAMRPKTEYTILAPVNTAFSN

EVTSTDRNLLRTILENHILKLKITLSELYNGQLLETLAGKLLRVFIYRT

AVCIENACMLRGSKESSRSALHIMRSIIKPADKSIYQLLIKDGRFK

IFLSLMEVAGLTDLLKQEGSYTVFAPTDEAFDGLTREDLALLKS

DVNTLRIILLYHFSNGIFINGGLEGGVTNMLKTLQGNNLRVFFV

ALIGLSLLTSGLWQVNNTIQVNSVDVPTSDLMATNGVIHVVKNVLYP

FLLKKLIRFIHIK

FVPGYRYIELPLTFLSKCSSGISISY

YFMMKAINSCPYLFFLHTAPEVTLTRVIEGEPSVSTFTKVIEEKPTITEITRVIEGEPSVSTFTKVIEGKPTITKITRIHEMEPTITKVVVEGDPLITKLTKVIEGQPSSVQFTRVIEGKGQE

**>Tr_PNprtl.loc01__N.00000152208 (NEWSINFRUP00000152208) [Takifugu rubripes]**

AAFALFVLSMFDKADSSAYDKIVAHSRIRARKE

GPNVCALQQVMGTKKYFSTCRNWYNKSICGRKA

TVLYECCPGYMKLEGMHGCPA

VAPIDDVYGTLGVVKATSTQRYSEISKLRPEIEGSGSYTFFAPSNEAWDSLDD

TMKNALVSNVNVELYNALHYHMSKRRLLTKDLKNGMTVPSMNLDLDLYINHYPNG

VVTVNCARIIHGNQVATNGVVHVIDRVISAVGNTIQDVIENDDELSTLS

DVAQSAGVLEKLGQPGQYTLFAPTNKAFEGLGQEVLERLRGDKGALK

ALVNFHLLDSVQCSEAIMSGTSYETMEGSNVIIGCDGESLTVNGIKMVLKKDIVTTNGVIHLIDKVLMPDS

AKQVMELVGDSQSTFRDMVAQLGLSSAMRPKAEYTLLAPHNDAFS

DEVMSKDQRLLRILLENHILKNKVVLGELYNGQHLETIGGKLLRVFIYRT

AVCIENSCLIRGSKEGINGALHITKTLLKPSEKTIYEILMENGRFK

IFLSLMEAAEMTDLLKQEGDFTLFAPSDEAFAGLTQSDLTLLKS

DITALRTILLYHFNKGIFIGGGLENGVTNLFKSLQGNNIKVIF

ANKTMQVNSLQIPESDIMGKNGVIHFVNHILYPG

DIPVGSQSLLSLLRKMITNIQIKVQQNLTTK

**>Tr_PNprtl.loc02__N.00000144260 (NEWSINFRUP00000144260) [Takifugu rubripes]**

MNPLLPVTFVLVALCSLASVDSSAYDKIVSHSRIRARKE

GPNVCALQQVMGTKKKYFSTCRNWYKGSICSKKA

VVLYECCPGYMKLEGMRGCPA

VAPIDHVYGTLGLLHATSTQQYAQMAGMREGLEGKGSFTMFAPSDDAWAQLDP

AVRASLESNVNVELKNALHFHMVNRRLLTKDLKNGISISSMYNNLGLYINHYSNG

IVTVNCARIVHGNQVATNGVVHVIDRVISAVGSTIQDVLDVDDELSSFSV

PLASGTMDKLGRPGQYTLFAPTNEAFNRLSPGHLERMMGNKNVIA

ALVNFHLLNSAQCSEAIMAGSVFETAEGSTVEIGCDGDSLTVNGIKMVLKKDIVTTNGVIHLIDRVLIPDS

AKEGLELIGESQKTFSNMVSEVGLAAAMRPKTEYTILAPVNSAFS

NEVMSTDRRLLRTILENHILKLKITLSELYSGQLLETLAGKLLRVFIYRT

AVCIENACMLRGSKEGSNSALHVMRSIIKPADKTIYQLLLLDGRFK

IFLSLMEVAGLTGLLKQEGSYTIFAPTDEAFDGLTPSDFELLKS

DVNALRVILLYHFSNGIFINGGLEGGVTNMLKTLQGNNLHVFS

VNNTIQVNSVDVPTSDLMATNGVIHVVKNVLYPP

DLPVGREDLLVLLKKLIRFIHIK

FVSGYRYVEIPLTFL

IPEETKFTRVIDGQSSVKTFTKVIEGEPTITKITRVIETEPTLTRVVVEGEPSITTLTRVIESQPSITQVTRVIEGE

### Periostin and periostin homolog sequences (all partial) for *C. milii*, *B. floridae*, and *P. marinus* (see Additional file 6).

**>Cm_PNprtl [Callorhinchus milii]**

VFTSFDQAESSYYDKIVTHSRIRAKTKG

PNVCALQQVVGTKKKYFSTCRNWYRKSICGKKA

VSYECCPGYSKVHAQKGCPA

PSVAPIDHVYSTLDIVGATTTQEYSDRSNLRKEIEGIGSFTFFAPSNEAWTILDS

EVRNALLSNVNIELLNALHYHMVNKRLMTKDLKNGLTLQSMHDDQTLLINHYSNGV

VTVNCARILLANQIATNGVVHVIDRVITAVGYTIEDFIENDEELLSL

TASGVLAKLGKKGHYTLFAPTNAAFDKLPQDVLQRILSDPVAL

LTALLNYHILESVQCSEAIMSGSAYTTLEGSNIEIGCDGDSLTINGKKMVNRKDIVTTNGVIHLIDEVLIPDAGK

ESIMALDQRMLKVILQNHVLKPKVALSELFNEQKLQTLGGNYLRVFVYRTV

IFLSLMESADLLDLLIAEGSWTLFVPTDDVFESISPDEMEQLKS

NKNALRHILMYHLLKGVFIGGGIEYGVTNILKSYQGSRVMV

VNETLLVNGLKSKESDIMANNGVIH

HFVQRLSHPEDLPNASQGLGTSSRR

RITNFIWGNYWPGYQETEMPLSF

**>Cm_TGFBIprtl [Callorhinchus milii]**

PNVCAMQKVMGVNKKYFTNCKQWYQRKVCGKST

VVSYECCPGYSKVHAQKGCPAG

ALPITNIYNTLRNVGSSTTQLYADRANLRPLIEGPGSYTFFAPSNEAWSSL

EIIDALVSNVNIELLNALQYHMVERRILTDELKHGTVLTSMYQELNVYIHHYPNGV

VHVIDRVITAITNTIQQTIDVDDSFENLR

AAVAAAGLSDLLDSEGQYTLFAPTDEAFRKVPPATLNRILGDPQALK

MLNYHILKNVQCAEAIISGSPMETLEGKPLEVGCITDSITLNDKSIVTDKDILATNGVIHKINELLIPDSGK

LISHLEGTEAVTLLAPQNEALQGK

DTSTVVNDDVRDLLLSHVVKGQAFSRNMYHGQVLETMNGKQLRVFVYRN

**>Bf_PN/TGFBI-prtl [Branchiostoma floridae]**

MRPLVLLGLAAVLQVAYAQVGYNVLYNWRDKVHDQG

PNVCAVEVVEDTNKQYWTSCRTFWNRQICERQT

MIRYECCTGYGEIPGEKGCT

TELPLKNIPATLEDTGADTMLLYTTRAGLTDQLENEGAFTVFAPSNEAWAELP

QEVIDALVSNVNVELRNALLYHMAEGVEEINDFANDRMLRTMYRNSPMRINNYADS

IYTVNCGRVLRPNQRATNGIVHIIDKVVTPTTGTIDDFVSNNDRFATL

RTAITAAGLSEAIQGEGPFTLFAPTNEAFNKIPPETLNRILGDADSLD

ALLKYHILDYGFCSAFITRRHKLRTMEGSRVNVSCGRG

EFRVNQEVKIVESDIVTDNGIIHVIDSVLIPNA

AKNVAEMSETVAPPKINEYLSSSGLIDEMAEGGPWTILAPTDQAFEGE

SDTAFQKEVLSRHIVEGQLTGRKLIEGQELQSIDGETTLRTSLYRK

TFCIDNACAYKGDHEANNGAVILIDKVLLPAEGSLWDLINQDGSF

STLKTAIQTAGLVDVFKNPAVFTVFAPTNEAFENLPAGTLDELLASSEDLTNLLQYHVVGQMWHRKAMRPNIKYRLPSV

QGRKLEMIRAKEEGSNEYVVTINGKAKITQGETLATNGLLHVIDQVLIPGQKGSANGEGDGEGGNNL

ISVINGGRETIGGSAGEGGSVSVSVGEGAGGEGSVIVGEGEGASNVVTGDGGPRVIKVTSRVTLPGSGPSIWDSIDQDADF

SILKSAISTAQLEDMLDRPGTYTFFAPTDRAFNALPEDALPNLLANPEQLSEILE

YHVVENVLNSKAVSPGVAIPLATLQGGQVTVRNVNGVVTVNSATVERADLINTNGIIHVIDKVLIP

**>Pm_PN/TGFBI-prtl_DW022362(2-886) [Petromyzon marinus]**

LVPVRNSRDIVDPRVRWEPRTMLASCLLAALVLSLVADASHFDRTVSHSRIRGRTQGANV

CAVQKVVDTEKKYYSNCKQWYQKQICGKKTIVTYECCPGYVRVDGVDGCSAITPIVNVYE

TLEPIEATLTQKYSNQSGLRPEIEGPGAHTMFAPSNEAWLELPKEVRDSLTTNVNIELLN

ALRYHMVDRRLLTSDLKDGTVLTSMYEKQKLYVNHYPNGIITMNCARLIRPNHLATNGVV

HVIDRVVVPVSNQIGRCHQLRRGHGVDAGRAVEASRTDAICFNFGRGPIHSLSVP

**>Pm_PN/TGFBI-prtl_DW021408(2-841) [Petromyzon marinus]**

LVPVRNSRDIVDPRVRLDGPGAYTFFMPTNEAWERIPAVQAAELEDPAVLKSLLLSHAVN

KRLLSRHLKSGSALASMQSGRSIYVQQSPNGILTVNCVRVIKPNILATNGVLHMVDGIIP

RVSNNIRKILEEDSELSTLKLAIQKAGLMDMVTKEGPITLLAPTNAAFEKVPVDVMERIL

KDPTSLKALVTHHIIRTVHCSEAILSGSSVDTVEGSSLVIGCDGDRLTINAEAVIEQKDI

VATNGVVHKVSEVFIPDLAKTAIELTQTEDLSKLLQHVF

**>Pm_PN/TGFBI-prtl_FD721541(1-855) [Petromyzon marinus]**

PGLRSELFRMECKWSTGFAAFLVLWLVHNASSSAIDRLLTHSRIRAIKEGPNMCAVQQIE

NGKKYFSSCQLWYRKKICNRPATVSYECCPGYVSLSGQTGCPAIVPLNSIYKTVDGLGIK

VTKARVDKADLGSDLDGPGAYTFFMPTNEAWERIPAALAAELEDPAVLKSLLLSHAVNKR

LLSRHLKSGSALASMQSGRSIYVQQSPNGILTVNCVRVIKPNILATNGVLHMVDGIIPRV

SNNIRKILEEDSELSTLKLAIQKAGLMDMVTKEGPITLLAPTNAA

**>Pm_PN/TGFBI-prtl_FD706478(3-830) [Petromyzon marinus]**

EAGLKSSLKVGEEYTILAPKDSAMEDSTISTSSDSTRKMLLNHVIRGRHLSNQLYHGQKL

ETLGGQQLRVFLYHRAICIETSCVNVRDKSTNTGALFVLDKVIKPATSTVMELLRNDARF

STLLSLVNVAGMAQKFGRQGEMTLFAPTNEAFKSLGPAELSKLKRNRKELAALINGHISQ

GILVSGGIVPVGSNLVKTAQGTRLDVIVRNGTTYVNNKKMVQADILGVNGVIHAIDGVIL

PGAQWTNAMVNDRPMTDEQLAIFQRLTGRKHTLKPK
