# Supplementary material for: Periostin shows increased evolutionary plasticity in its alternatively spliced region
Source: BMC Evol Biol. 2010 Jan 28;10:30. doi: 10.1186/1471-2148-10-30 (PMC2824660; doi:10.1186/1471-2148-10-30)
Supplement: Additional file 6 — Additional evolutionary and phylogenetic considerations. This document summarizes additional evolutionary and phylogenetic considerations regarding periostin and its paralog, TGFBI, based on analyses of periostin/TGFBI homologs outside the euteleostomes. Contains Supplementary Figures S4 - S7. [file 1471-2148-10-30-S6.PDF]

Additional file 6, text with Figures S4 – S7

Evolutionary and phylogenetic considerations regarding periostin and its paralog, TGFBI

According to our survey of the SMART domain database (http://smart.embl-heidelberg.de/, data not shown), periostin and TGFBI – with their sequence of secretion signal, EMI domain and tetrad of FAS1 domains – represent one of only two vertebrate contexts for FAS1 domains, the second being the presence of FAS1 domains in the scavenger receptors stabilin 1 and 2 (STAB1 and STAB2, also FEEL-1/2 and FELE-1/2), where as many as seven FAS1 domains occur interspersed with other domains.

Outside the vertebrate group, candidate genes encoding the domain architecture "signal sequence – EMI domain – 4x FAS1 domain" can additionally be found in cephalochordates (lancelet, *Branchiostoma floridae*; RefSeq:XP_002235318.1) and gastropods (California sea hare, *Aplysia californica*; Uniprot:Q8N0B2_APLCA). Thus it appears that this domain architecture predates the protostome / deuterostome split within the Coelomata.

This picture is muddled by a lack of obvious candidates for four-fold FAS1 domain proteins within deuterostome groups of varying proximity to the vertebrates: the comparatively well studied Tunicates and Echinoderms (represented by the genome assemblies for *Ciona intestinalis* and the sea urchin *Strongylocentrotus purpuratus*) do not appear to have them, even without EMI domain. We are assuming that this is due to a secondary loss in these groups, given that the four-fold FAS1 domain structure per se is phylogenetically very broadly encountered, although not ubiquitous. It was originally described in grasshopper (*Schistocerca americana*) and fruit fly (*Drosophila melanogaster*) [23] for the protein fasciclin (which provided the name for the FAS1 (fasciclin I) domain), and is generally common in arthropods. According to records in the SMART database, there are even a few known instances of four FAS1 domain proteins in bacteria (curiously, all in marine and/or psychrophilic species) and in fungi.

Given the framework outlined above, it was reasonable to hope that we might be able to phylogenetically pinpoint the periostin / TGFBI split and the appearance of the repeat structure for periostin, taking advantage of complete genome sequences from relevant species that have become available recently.

In this context, the elephant shark or ghost shark (*Callorhinchus milii*) was of interest as a cartilaginous fish and thus as representative of the Chondrichthyes, the non-euteleostome branch of the jawed vertebrates (Gnathostomata). We found evidence supporting the presence of both periostin and TGFBI in the elephant shark genome (see Figure S4).

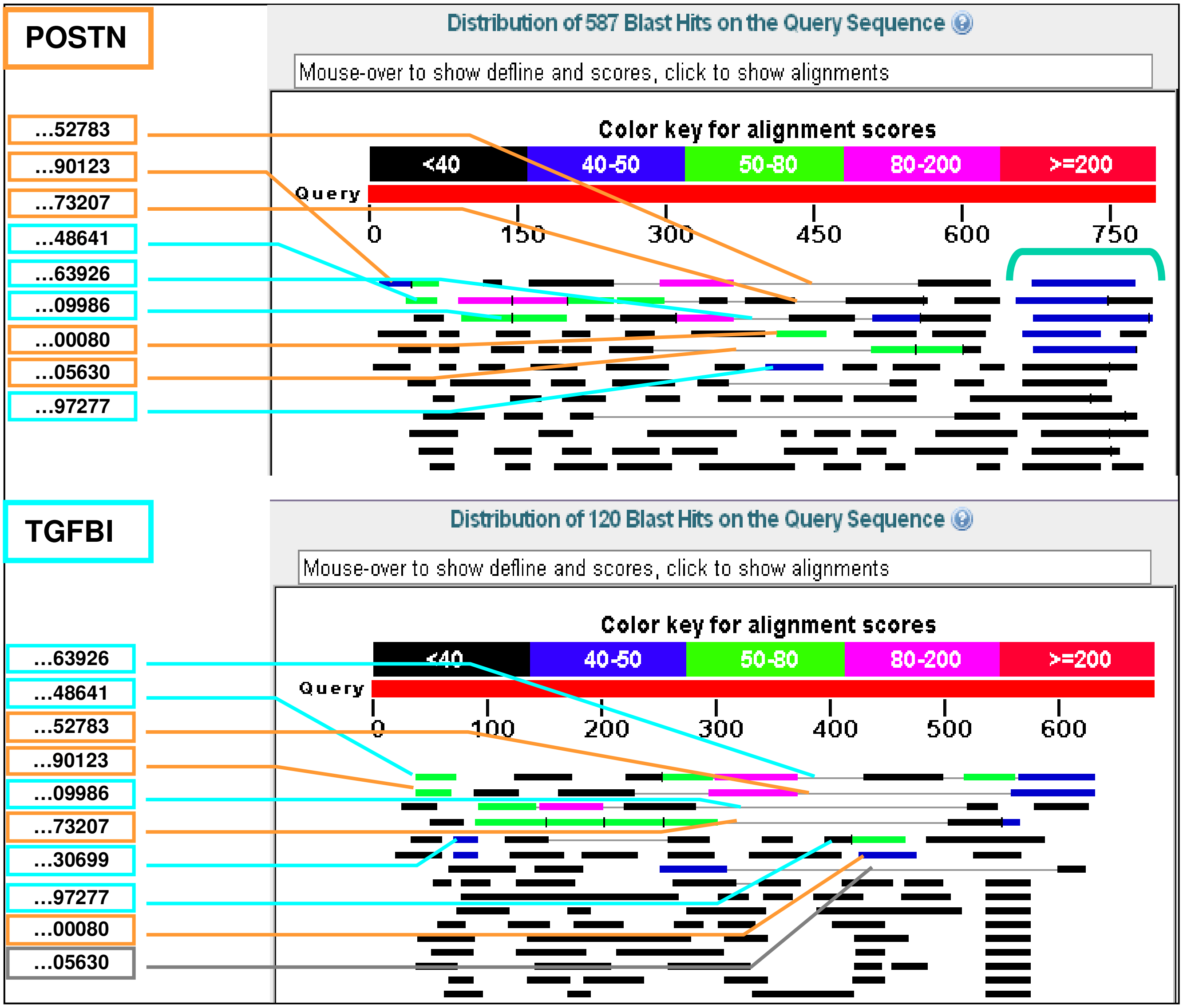

Figure S4: Graphical representation of the disambiguation process for separating POSTN and TGFBI in the elephant shark (*Callorhinchus milii*) genome (unfinished assembly).
TBLASTN searches were performed against *C. milii* "whole genome shotgun" sequences using the "genomic BLAST" server at http://www.ncbi.nlm.nih.gov/sutils/genom_table.cgi?organism=euk with *Danio rerio* (locus 1) periostin protein sequence (top panel) and TGFBI protein sequence (bottom panel) as queries. *C. milii* subject sequences (identified by the last five digits of their sequence accession number) were annotated with regard to being primary or secondary hits for the respective search. It emerged that by this metric alone, subject sequences, while commonly appearing in both searches, could be near-unambiguously assigned to either POSTN or TGFBI (reflected in the color coding, orange for POSTN, cyan for TGFBI). This, in turn, enabled the partial reconstruction of *C. milii* POSTN and TGFBI sequences. The much higher number of hits for the POSTN compared to the TGFBI query (587 vs. 120) is mostly due to the POSTN C-terminal region (blue-green bracket), which results in a disproportional number of hits.

This is borne out in an alignment-based phylogenetic tree with the partially reconstructed sequences (Figure S5), where the candidate periostin and TGFBI sequences from shark co-cluster with their respective euteleostome orthologs.

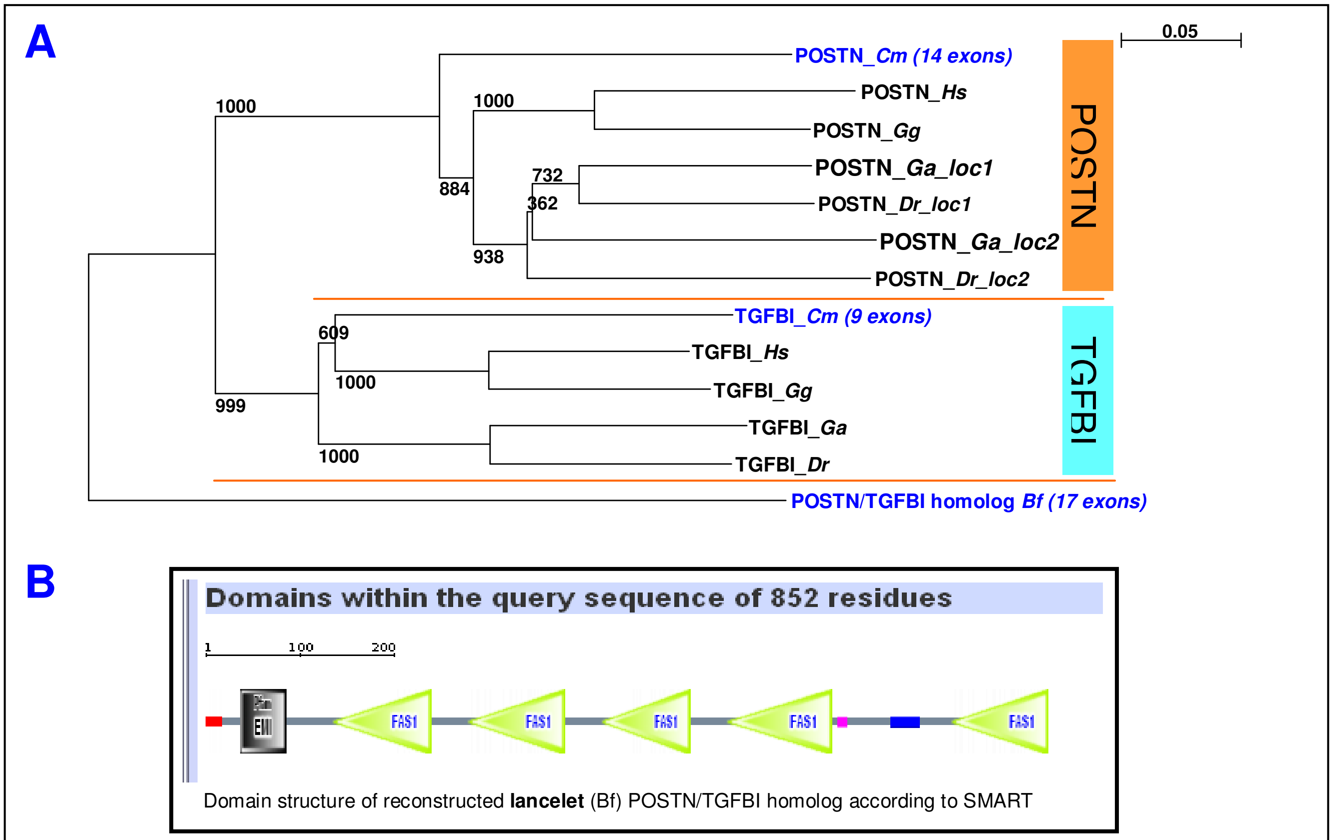

C **Abbreviated taxonomic lineages:**

Hs, Gg, Dr, Ga:
Coelomata; Deuterostomia; Chordata; Craniata; Vertebrata; Gnathostomata; Euteleostomi …

Cm: Coelomata; Deuterostomia; Chordata; Craniata; Vertebrata; Gnathostomata; **Chondrichthyes**; Holocephali; Chimaeriformes; Callorhinchidae; ***Callorhincus milii***

Bf: Coelomata; Deuterostomia; Chordata; **Cephalochordata**; Branchiostomidae; ***Branchiostoma floridae***

Figure S5: A POSTN/TGFBI homolog in lancelet (*Branchiostoma floridae*).
(A) Phylogenetic tree based on a ClustalW alignment of POSTN and TGFBI sequences from select euteleostome species, the reconstructed POSTN and TGFBI sequences from elephant shark (*C. milii*, see Fig.S4), and reconstructed POSTN/TGFBI homolog from the lancelet *B. floridae* (based on predicted lancelet protein Refseq:XP_002235318.1 and translated lancelet ESTs), which clearly falls outside the POSTN and TGFBI groups. Accession numbers for TGFBI sequences (except Cm): RefSeq:NP_000349.1 (Hs), RefSeq:NP_990367.1 (Gg), RefSeq:NP_878282.1 (Dr), Ensembl:ENSGACP00000021567 (Ga).
(B) Domain structure of the reconstructed lancelet POSTN/TGFBI homolog (as predicted by SMART, http://smart.embl-heidelberg.de/), which interestingly features a fifth C-terminal FAS1 domain. Note that the tree topology given in (A) is not attributable to this fifth domain, since the tree was calculated excluding gapped columns.
(C) Taxonomic lineages of the newly considered species *C. milii* and *B. floridae*.

However, the question if elephant shark periostin also has the repeat structure universally encountered in euteleostomes could not be resolved: While we identified multiple candidate repeat regions with a periodicity of 13 amino acids and similarity to a periostin repeat consensus based on teleost fish (Figure S6), the fragmentary nature of the elephant shark genome assembly made it impossible to establish or rule out an actual inter-exon connection between matches to repeats and those to other regions of the periostin gene.

A genome-based reconstruction of the sequence for the candidate periostin/TGFBI homolog from lancelet, a cephalochordate, and its alignment with periostin and TGFBI sequences showed it falling outside the periostin and TGFBI clusters in the resulting dendrogram (Figure S5).

A

Best repeat match in elephant shark:

```
> AAVX01225691.1 <http://blast.fugu-sg.org/cgi-bin/scaff_shark.pl?scaffold=AAVX01225691.1>
         Length = 1839

 Score =  109 bits (240), Expect(3) = 3e-41
 Identities = 65/145 (44%), Positives = 78/145 (53%)
 Frame = -2

Query: 1    PSITKVTRVIEGEPSITKVTRVIEGEPSITKVTRVIEGEPSITKVTRVIEGEPSITKVTR 60
            PS     T++ E EPS     TR+ E EPS     TR+ E EPS      R+ E EPS     TR
Sbjct: 1742 PSRVAETKIREMEPSRVAETRIREMEPSRVAETRIREMEPSRVAEIRIREMEPSRVAETR 1563

Query: 61   VIEGEPSITKVTRVIEGEPSITKVTRVIEGEPSITKVTRVIEGEPSITKVTRVIEGEPSI 120
            + E EPS     T++ E EPS     TR+ E EPS     TR+ E EPS     TR+ E EPS
Sbjct: 1562 IWEMEPSRVAETKIREMEPSRVAETRIREMEPSRVAETRIREMEPSRVAETRIREMEPSG 1383

Query: 121  TKVTRVIEGEPSITKVTRVIEGEPS 145
               TR+ E EPS     TR+ E EPS
Sbjct: 1382 VAETRIREMEPSRVAETRIREMEPS 1308
```

B

Shark repeat consensus (11 sequences)

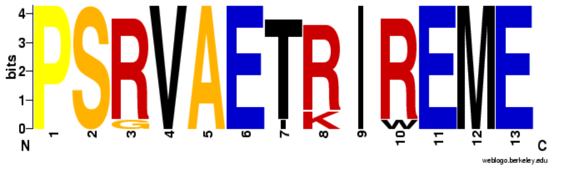

Teleost fish repeat exon 18=19 consensus (45 sequences)

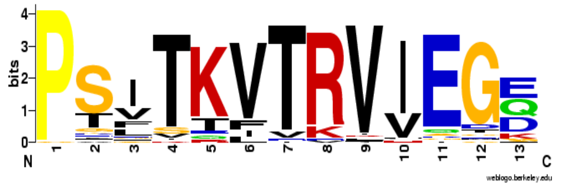

Figure S6: Best TBLASTN hit in the *C. milii* genome for a query of concatenated teleost fish repeat consensus sequences.
(A) A query of concatenated teleost fish repeat consensus sequences (based on 45 repeat units from different teleost fish species, see main text for details) was used as a query in a TBLASTN search against the elephant shark genome sequence (via http://www.ncbi.nlm.nih.gov/sutils/genom_table.cgi?organism=euk); the BLAST alignment for the best hit is shown .
(B) A comparison of the repeat consensus from this hit (top) to the teleost repeat consensus used for the TBLASTN search show (bottom) via sequence logo representation.

So far, these findings are consistent with the prevalent concept of whole genome duplication events within the chordates that provide the most straightforward explanation for the emergence of the periostin / TGFBI paralog pair: Apart from the most recent whole genome duplication (WGD) event at the base of teleost fish, two other WGD events, one at the base of the jawed vertebrates and another at the base of all vertebrates, are thought to have occurred [70].

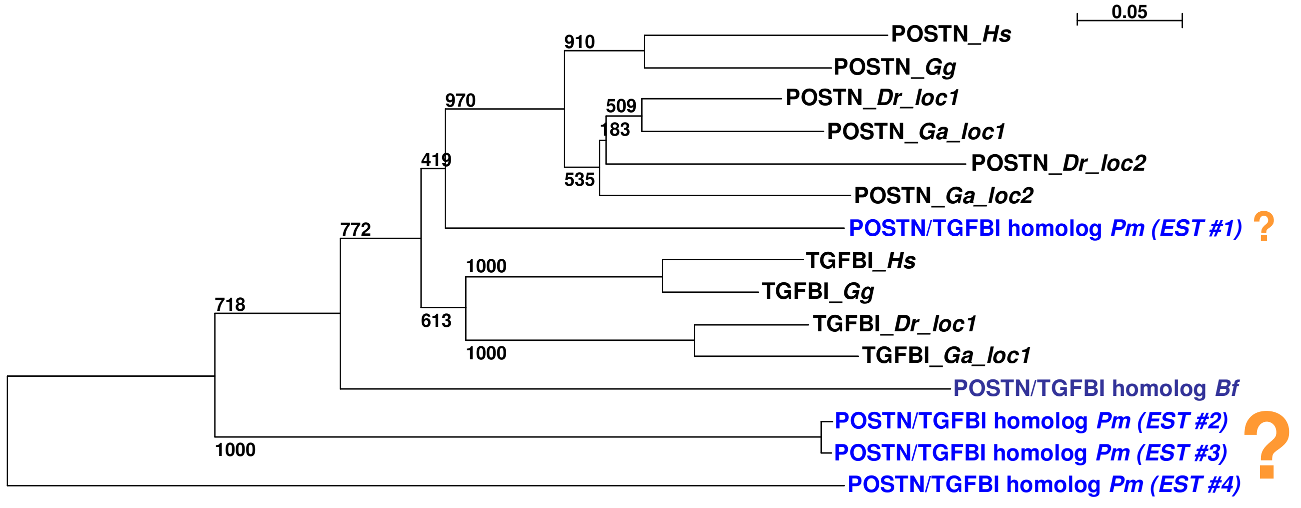

Abbreviated taxonomic lineages:

Hs, Gg, Dr, Ga:
Coelomata; Deuterostomia; Chordata; Craniata; Vertebrata; Gnathostomata; Euteleostomi …

Cm: Coelomata; Deuterostomia; Chordata; Craniata; Vertebrata; Gnathostomata; **Chondrichthyes**; Holocephali; Chimaeriformes; Callorhinchidae; ***Callorhinchus milii***

Pm: Coelomata; Deuterostomia; Chordata; Craniata; Vertebrata; **Hyperoartia**; Petromyzontiformes; Petromyzontidae; ***Petromyzon marinus***

Bf: Coelomata; Deuterostomia; Chordata; **Cephalochordata**; Branchiostomidae; ***Branchiostoma floridae***

Figure S7: Phylogenetic tree based on the alignment of select POSTN and TGFBI protein sequences and four translated EST sequences from putative lamprey (*Petromyzon marinus*) POSTN/TGFBI homologs. EST #1 groups with POSTN, ESTs #2, #3, #4 form an outgroup relative to all other sequences, including the POSTN/TGFBI homolog from lancelet, which is contrary to the lamprey's phylogenetic position relative to euteleostomes and cephalochordates (see taxonomic lineages below). The interpretation of this result is difficult (see main text for details).
P. marinus EST accession numbers (and translated nucleotide ranges): EST #1: GenBank:DW022362 (2-886), EST #2: GenBank:DW021408 (2-841), EST #3: GenBank:FD721541 (1-855), EST #4: GenBank:FD706478 (3-830).

Crucially, searches in the genome sequence of the lamprey (*Petromyzon marinus*), a jawless vertebrate, and as such positioned after the first chordate WGD event affecting vertebrates, but not subject to the second one at the base of the jawed vertebrates, produced complex and ambiguous results (see Figure S7). Their interpretation was substantially hampered by the extremely preliminary status of the genome assembly.

Consequently, while successfully fitting periostin and TGFBI into the larger framework of chordate evolution, we are presently unable to precisely identify the WGD event responsible for the split between these two genes and to clarify the origin of periostin's C-terminal repeat region beyond the euteleostome group.
